# Neonatal mortality risk of vulnerable newborns: A descriptive analysis of subnational, population‐based birth cohorts for 238 203 live births in low‐ and middle‐income settings from 2000 to 2017

**DOI:** 10.1111/1471-0528.17518

**Published:** 2023-05-08

**Authors:** Elizabeth A. Hazel, Daniel J. Erchick, Joanne Katz, Anne C. C. Lee, Michael Diaz, Lee S. F. Wu, Keith P. West, Abu Ahmed Shamim, Parul Christian, Hasmot Ali, Abdullah H. Baqui, Samir K. Saha, Salahuddin Ahmed, Arunangshu Dutta Roy, Mariângela F. Silveira, Romina Buffarini, Roger Shapiro, Rebecca Zash, Patrick Kolsteren, Carl Lachat, Lieven Huybregts, Dominique Roberfroid, Zhonghai Zhu, Lingxia Zeng, Seifu H. Gebreyesus, Kokeb Tesfamariam, Seth Adu‐Afarwuah, Kathryn G. Dewey, Stephaney Gyaase, Kwaku Poku‐Asante, Ellen Boamah Kaali, Darby Jack, Thulasiraj Ravilla, James Tielsch, Sunita Taneja, Ranadip Chowdhury, Per Ashorn, Kenneth Maleta, Ulla Ashorn, Charles Mangani, Luke C. Mullany, Subarna K. Khatry, Vundli Ramokolo, Wanga Zembe‐Mkabile, Wafaie W. Fawzi, Dongqing Wang, Christentze Schmiegelow, Daniel Minja, Omari Abdul Msemo, John P. A. Lusingu, Emily R. Smith, Honorati Masanja, Aroonsri Mongkolchati, Paniya Keentupthai, Abel Kakuru, Richard Kajubi, Katherine Semrau, Davidson H. Hamer, Albert Manasyan, Jake M. Pry, Bernard Chasekwa, Jean Humphrey, Robert E. Black, Subnational Collaborative Group for Vulnerable Newborn Mortality, Rolf D. W. Klemm, Allan B. Massie, Maithilee Mitra, Sucheta Mehra, Kerry J. Schulze, Alfred Sommer, Md. Barkat Ullah, Alain B. Labrique, Mabhubur Rashid, Saijuddin Shaikh, Nazma Begum, Nabidul Haque Chowdhury, Md. Shafiqul Islam, Rasheda Khanam, Dipak Kumar Mitra, Abdul Quaiyum, Modiegi Diseko, Joseph Makhema, Yue Cheng, Meselech Roro, Bilal Shikur Endris, Charles D. Arnold, Rajiv Bahl, Nita Bhandari, Jose Martines, Sarmila Mazumder, Lotta Hallamaa, Juha Pyykkö, Willy Urassa, Phillippe Deloron, Ib Christian Bygbjerg, Sofie Lykke Moeller, Thor Grundtvig Theander, Alfa Muhihi, Ramadhani Abdallah Noor, Moses R. Kamya, Miriam Nakalembe, Godfrey Biemba, Julie M. Herlihy, Reuben K. Mbewe, Fern Mweena, Kojo Yeboah‐Antwi, Andrew Prendergast, Vulnerable Newborn Measurement Core Group, Joy E. Lawn, Hannah Blencowe, Eric Ohuma, Yemi Okwaraji, Judith Yargawa, Ellen Bradley, Lorena Suarez Idueta

**Affiliations:** ^1^ International Health Department Johns Hopkins Bloomberg School of Public Health Baltimore Maryland USA; ^2^ Pediatric Newborn Medicine Brigham and Women's Hospital, Harvard Medical School Boston Massachusetts USA; ^3^ Department of International Health, Center for Human Nutrition Johns Hopkins Bloomberg School of Public Health Baltimore Maryland USA; ^4^ BRAC JP Grant School of Public Health Dhaka Bangladesh; ^5^ JiVitA Maternal and Child Health Research Project Rangpur Bangladesh; ^6^ Department of International Health Johns Hopkins Bloomberg School of Public Health Baltimore Maryland USA; ^7^ Child Health Research Foundation Dhaka Bangladesh; ^8^ Projahnmo Research Foundation Dhaka Bangladesh; ^9^ Post‐Graduate Program in Epidemiology – Federal University of Pelotas Pelotas Brazil; ^10^ Harvard T.H. Chan School of Public Health Boston Massachusetts USA; ^11^ Beth Israel Deaconess Medical Center Boston Massachusetts USA; ^12^ Department of Food Technology, Safety and Health Ghent University Ghent Belgium; ^13^ Poverty, Health and Nutrition Division International Food Policy Research Institute Washington District of Columbia USA; ^14^ Namur University Namur Belgium; ^15^ Belgian Health Care Knowledge Centre Brussels Belgium; ^16^ Department of Epidemiology and Biostatistics, School of Public Health Xi'an Jiaotong University Health Science Centre Xi'an China; ^17^ Department of Nutrition and Dietetics, School of Public Health Addis Ababa University Addis Ababa Ethiopia; ^18^ Department of Food Technology, Safety, and Health, Faculty of Bioscience Engineering Ghent University Ghent Belgium; ^19^ Department of Nutrition and Food Science University of Ghana Accra Ghana; ^20^ Department of Nutrition, Institute for Global Nutrition University of California Davis California USA; ^21^ Kintampo Health Research Centre Kintampo Ghana; ^22^ Research and Development Division Ghana Health Service Accra Ghana; ^23^ Columbia University's Mailman School of Public Health New York New York USA; ^24^ Aravind Eye Hospital Madurai India; ^25^ George Washington University Milken Institute School of Public Health Washington District of Columbia USA; ^26^ Centre for Health Research and Development Society for Applied Studies New Delhi India; ^27^ Faculty of Medicine and Health Technology Tampere University and Tampere University Hospital Tampere Finland; ^28^ School of Global and Public Health Kamuzu University of Health Sciences Blantyre Malawi; ^29^ Faculty of Medicine and Health Technology Tampere University Tampere Finland; ^30^ NNIPS Kathmandu Nepal; ^31^ HIV and Other Infectious Diseases Research Unit South African Medical Research Council Cape Town South Africa; ^32^ Gertrude H Sergievsky Center, Vagelos College of Physicians and Surgeons Columbia University Irving Medical Center New York New York USA; ^33^ Health Systems Research Unit South African Medical Research Council Cape Town South Africa; ^34^ South African Research Chair in Social Policy at College Graduate of Studies University of South Africa Pretoria South Africa; ^35^ Department of Global and Community Health, College of Public Health George Mason University Fairfax Virginia USA; ^36^ Centre for Medical Parasitology Department of Immunology and Microbiology, University of Copenhagen, and Department of Infectious Diseases, Copenhagen University Hospital Copenhagen Denmark; ^37^ National Institute of Medical Research Tanga Tanzania; ^38^ Department of Global Health Milken Institute School of Public Health Washington District of Columbia USA; ^39^ Ifakara Health Institute Dar es Salaam Tanzania; ^40^ ASEAN Institute for Health Development Mahidol University Salaya Thailand; ^41^ College of Medicine and Public Health Ubon Ratchathani University Ubon Ratchathani Thailand; ^42^ Infectious Diseases Research Collaboration Kampala Uganda; ^43^ Ariadne Labs Brigham and Women's Hospital and Harvard T.H. Chan School of Public Health Boston Massachusetts USA; ^44^ Division of Global Health Equity, Department of Medicine Brigham and Women's Hospital Boston Massachusetts USA; ^45^ Department of Medicine Harvard Medical School Boston Massachusetts USA; ^46^ Department of Global Health Boston University School of Public Health Boston Massachusetts USA; ^47^ Section of Infectious Diseases, Department of Medicine Boston University Chobanian & Avedisian School of Medicine Boston Massachusetts USA; ^48^ University of Alabama at Birmingham Birmingham Alabama USA; ^49^ Centre for Infectious Disease Research in Zambia Lusaka Zambia; ^50^ Zvitambo Institute for Maternal and Child Health Research Harare Zimbabwe

**Keywords:** low‐and middle‐income countries, obstetrics and gynaecology, paediatrics: neonatal, preterm, small‐for‐gestational age

## Abstract

**Objective:**

We aimed to understand the mortality risks of vulnerable newborns (defined as preterm and/or born weighing smaller or larger compared to a standard population), in low‐ and middle‐income countries (LMICs).

**Design:**

Descriptive multi‐country, secondary analysis of individual‐level study data of babies born since 2000.

**Setting:**

Sixteen subnational, population‐based studies from nine LMICs in sub‐Saharan Africa, Southern and Eastern Asia, and Latin America.

**Population:**

Live birth neonates.

**Methods:**

We categorically defined five vulnerable newborn types based on size (large‐ or appropriate‐ or small‐for‐gestational age [LGA, AGA, SGA]), and term (T) and preterm (PT): T + LGA, T + SGA, PT + LGA, PT + AGA, and PT + SGA, with T + AGA (reference). A 10‐type definition included low birthweight (LBW) and non‐LBW, and a four‐type definition collapsed AGA/LGA into one category. We performed imputation for missing birthweights in 13 of the studies.

**Main Outcome Measures:**

Median and interquartile ranges by study for the prevalence, mortality rates and relative mortality risks for the four, six and ten type classification.

**Results:**

There were 238 203 live births with known neonatal status. Four of the six types had higher mortality risk: T + SGA (median relative risk [RR] 2.6, interquartile range [IQR] 2.0–2.9), PT + LGA (median RR 7.3, IQR 2.3–10.4), PT + AGA (median RR 6.0, IQR 4.4–13.2) and PT + SGA (median RR 10.4, IQR 8.6–13.9). T + SGA, PT + LGA and PT + AGA babies who were LBW, had higher risk compared with non‐LBW babies.

**Conclusions:**

Small and/or preterm babies in LIMCs have a considerably increased mortality risk compared with babies born at term and larger. This classification system may advance the understanding of the social determinants and biomedical risk factors along with improved treatment that is critical for newborn health.

## 1 INTRODUCTION

In 2020, 2.4 million babies died during the first month after birth, and over three‐quarters of these deaths occurred in two regions – sub‐Saharan Africa (1.1 million deaths) and Southern Asia (0.9 million deaths).^1^ Neonatal deaths (deaths that occur within 28 days after birth) have decreased in the past decades, from an estimated 45.6 deaths in 1990 to 27.1 deaths per 1000 livebirths in sub‐Saharan Africa and 57.1 to 23.2 deaths per 1000 livebirths in Southern Asia.^1^ Despite this progress, the mortality risk for neonates is unacceptably high and inequally distributed. The 2014 Every Newborn Action Plan set a mortality rate target of 12 or fewer neonatal deaths per 1000 livebirths and this target is now also a part of the 2030 Sustainable Development Goal (SDG).^2^, ^3^

There is elevated mortality risk associated with babies ‘born too early’ (preterm) or ‘born too small’ (small‐for‐gestational age [SGA]) or both. In prior analysis, we found babies born SGA are twice as likely to die in the neonatal period, preterm babies have seven times the mortality risk, and babies born both preterm and SGA have up to 15 times the risk.^4^ Sub‐Saharan Africa and Southern Asia combined include 81% of preterm and 72% of low birthweight (LBW) babies born globally.^3^, ^5^, ^6^

Historically, LBW (<2500 g) has been used to identify vulnerable newborns. LBW is caused by being preterm, having fetal growth restriction (FGR) or a combination of the two. As the underlaying aetiology of preterm and SGA is different, it is important to consider these separately because the outcomes and preventive interventions will differ as well. Additionally, babies born large‐for‐gestational age (LGA) (>90th centile compared with a standard population) have excess health risks.^7^ A more granular classification system is needed to identify and understand the different risks for vulnerable babies to effectively target interventions, policies and programmes.^8^

In countries with complete and high‐quality vital registration data systems, it is possible to estimate national‐level birth outcomes and associated neonatal mortality risks. In LMICs without these systems, we cannot empirically generate national estimates, but we can use population‐based subnational studies that collected high‐quality data on birth outcomes and neonatal mortality to estimate the associated neonatal mortality risks.

In this paper, we describe the neonatal mortality risks associated with four, six and ten vulnerable newborn type classifications based on combinations of size‐for‐gestational age, delivery at term or preterm, and low or not‐low birthweight (Table 1).^8^ The estimates presented in this analysis are intended only to describe the data available by study and should not be interpreted as global, regional or country‐level estimates.

**TABLE 1:** Key findings.

**1. What was known?**
Babies born preterm and/or small are at higher risk of dying during the neonatal period. Previously neonatal mortality was estimated for these conditions separately. However, these conditions can overlap and may have compounding mortality risks. Disease and mortality burden for preterm and/or small babies is higher in low‐and‐middle income countries (LMICs), also where data availability is the lowest.
**2. What was done that is new?**
We systematically searched and identified 16 studies from nine LMICs that collected high‐quality, population‐based data on birth outcomes with follow‐up through the neonatal period from 2000‐2017. Our pooled dataset of 238,143 livebirths provides the first multi‐country mortality estimates of these newborn types in LMICs. We defined and described the neonatal mortality risks for vulnerable newborn types categorized by preterm (PT) and term (T), size‐for‐gestational age (small (SGA), appropriate (AGA) and large (LGA)) and low birthweight (LBW) and non‐LBW (nLBW).
**3. What was found?**
*Preterm risks*: All preterm types had high neonatal mortality risk with PT + SGA as the highest risk (median relative risk (RR) 10.4, interquartile range (IQR): 8.6–13.9 by study). *Risks for babies born at term*: T + SGA had additional risk (median RR: 2.8, IQR: 2.0–3.2) and also the greatest prevalence (median: 25.0%, IQR: 18.8%–41.5%) of the vulnerable types, indicating the highest population mortality burden. T + LGA babies had no additional detected risk compared to T + AGA babies. *Usefulness of LBW categorization*: T + SGA babies who were also LBW had greater mortality risk (median RR 4.9, IQR: 3.1–6.4) compared to T + SGA babies who were nLBW (median RR 1.7, IQR: 1.4, 2.2). In settings with high T‐SGA prevalence, it may be programmatically important to track LBW as well.
**4. What next?**
*Action in preventive programmes*: This categorization of vulnerable newborn types provides more granular detail on mortality risks, useful for improving measurement, understanding the disease aetiology and epidemiology, and improving clinical care and population‐based interventions. *Research gaps*: High quality routine data systems that include gestational age, birthweight, and sex for every live‐and stillbirth with linked neonatal mortality data are needed to adequately track vulnerable newborn population level health.

## 2 METHODS

This is a secondary analysis of individual participant data from multiple studies; women and newborns did not have direct participation in this study (Table S1). We identified population‐based studies in LMICs that collected data on birthweight and gestational age at delivery for newborns born since 2000. Studies were identified through systematic review of peer‐reviewed literature databases, clinical trial registries and open data repositories and through professional networks. Further details of the study identification methods have been presented elsewhere.^9^ Principal investigators could send their de‐identified data for central processing or perform the analysis themselves with standard statistical code to assess the quality of the data, construct standardised study outcomes, and generate study‐specific estimates.

### 2.1 Inclusion and exclusion criteria

We defined study‐ and individual‐level exclusion criteria. To be included, studies must have sampled more than 300 live births, assessed gestational age at delivery through early ultrasound or timing of last menstrual period (LMP), collected data after the year 2000, and be population‐based including both home and facility births. Studies that sampled facility‐level births were included if 80% or more of the population delivered in a health facility. Studies that sampled from antenatal care (ANC) clinics were considered population‐based if 90% or more pregnant women received at least one ANC visit in the areas sampled.

Studies compiled for the prevalence paper that followed survival for at least 28 days after delivery were assessed for inclusion in the mortality analysis. Studies were excluded if (1) they had fewer than 20 neonatal deaths (the reduced sample size impeded investigation of mortality risk by type categorisation) or (2) data missingness was greater than 70% among neonatal deaths (combined gestational age at delivery, birthweight and infant sex). As missing type was primarily driven by missing birthweight, we imputed birthweight for studies with missing birthweight ranging from 10% to 70%.

Data quality of the included studies was assessed using proportion of missing or improbable birthweights, gestational age and missing sex. We excluded missing measured (or unable to impute birthweight due to missing covariates), gestational age, sex or a gestational age <22^+0^ weeks or >44^+6^ weeks for which it was not possible to assess size‐for‐gestational age. Birth records with implausible measured or imputed birthweights (<250 or ≥6500 g) or implausible combinations of measured or imputed birthweight and gestational age (defined as birthweight >5 standard deviations above the mean birthweight for gestational age and sex) were excluded. We also investigated heaping of birthweight (measured only) as a measure of the data collection quality. We calculated a heaping index by study defined as the number of births reported at exactly 2500 g divided by the number with 249 g below and above 2500 g. Lower values of this heaping index indicate higher quality data collection and documentation practices.

### 2.2 Description of recalibration and imputation methods

We imputed birthweight at the study level to calculate size‐for‐gestational age in 13 studies (Table S2). Eight of the 13 studies included infants with ‘birthweight’ measured in the early neonatal period. For these studies, we first recalibrated all infant weights to weight at the time of delivery based on a longitudinal model of daily weight measurements on newborns in the first 10 days. The longitudinal dataset was collected on a subset of infants enrolled in a clinical trial of chlorhexidine newborn cleansing from 2002 to 2005 in rural Nepal.^10^, ^11^ We then used these recalibrated birthweights multiply to impute missing birthweight based on maternal education, age and parity, single or multiple pregnancy, infant sex, gestational age and neonatal survival status. Additional details on the recalibration and imputation methods have been previously published by the authors.^12^

### 2.3 Exposure and outcome definitions

We categorised every included newborn based on gestational age at delivery (preterm birth <37 completed weeks [PT] or term ≥37 weeks [T]) and size‐for‐gestational age defined as SGA <10th centile; or LGA >90th centile or AGA between 10th and 90th centile using a modified version (extended to include GA from 22^+0^ to 44^+6^ weeks) of the INTERGROWTH‐21st international newborn size for gestational age and sex standards.^13^ Different combinations of these outcomes generate six mutually exclusive newborn types: T + AGA (reference), T + SGA, T + LGA, PT + SGA, PT + AGA and PT + LGA. We examined a four‐type classification that collapsed LGA/AGA: T + nonSGA (reference), T + SGA, PT + nonSGA and PT + SGA. Finally, we also generated a more complex classification (including LBW) for ten types including T + AGA + nonLBW (reference), T + LGA + nonLBW, T + AGA + LBW, T + SGA + nonLBW, T + SGA + LBW, PT + LGA + nonLBW, PT + LGA + LBW, PT + AGA + nonLBW, PT + AGA + LBW and PT + SGA + LBW. To estimate neonatal mortality risk, infant survival status was documented in each included study for the first 28 days (0–27 days) after delivery. Infants who were lost to follow‐up were censored.

### 2.4 Analysis

We calculated the proportion of births excluded from the analysis and reason for exclusion (i.e. missing or improbable data) by neonatal survival status in each study and described the demographic and obstetric characteristics. We calculated type prevalence, neonatal mortality rate (NMR), defined as the number of neonatal deaths per 1000 livebirths, crude relative risk ratios (RR) and 95% confidence intervals (95% CI). We reported these statistics for each study and then the overall median and interquartile range (IQR) by type. As these study level estimates were included in a global model of type prevalence and mortality risks and the analytical aim is descriptive, we did not perform meta‐analyses.^14^

## 3 RESULTS

**FIGURE 1:**
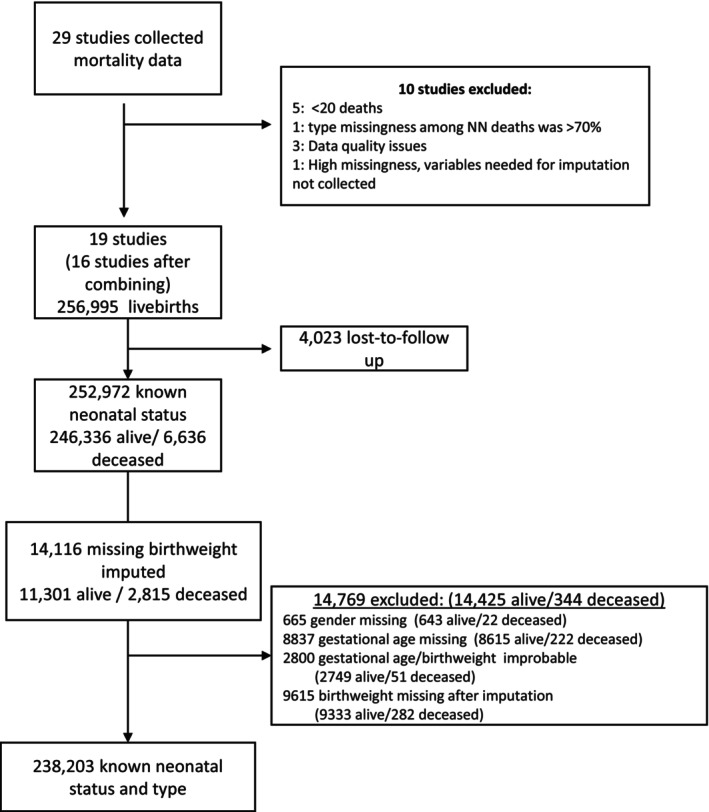
Flowchart of studies and live births included in the mortality analysis by type.

We identified 29 studies: five were excluded due to fewer than 20 neonatal deaths in the study, and five were excluded for other reasons, resulting in 19 studies (Figure 1).^15^, ^16^, ^17^, ^18^, ^19^, ^20^, ^21^, ^22^ In six studies from Burkina Faso, Malawi and one of the Tanzania studies, we pooled the data (two studies per country) that were carried out in the same site and by the same study teams, giving us 16 studies. We assigned study ID based on country and timing of the data collection (Table S2).

This analysis includes data from 16 subnational datasets from nine countries with data collected from 2000 to 2017 (Table S3).^23^, ^24^, ^25^, ^26^, ^27^, ^28^, ^29^, ^30^, ^31^, ^32^, ^33^, ^34^, ^35^, ^36^, ^37^, ^38^, ^39^, ^40^, ^41^ Seven studies were based in sub‐Saharan Africa, seven in Southern Asia, one in Eastern Asia and one in Latin America. Gestational age of the pregnancy was calculated from LMP collected during pregnancy for 11 studies, one study collected LMP during pregnancy and at delivery, two studies used ultrasound estimation, and two studies used a combination of ultrasound/LMP (Table S2). Neonatal mortality rates measured in the studies ranged from 8.7 deaths in Brazil to 45.1 deaths in Bangladesh per 1000 livebirths. Loss to follow‐up was minimal. In most studies, <5% were lost to follow‐up during the neonatal period, and in one study (Tanzania 2) 7.2% (Table S2). This subset of studies had a similar heaping index to the full set used in the prevalence analysis.^9^ The median heaping index was 6.6% (IQR 1.6%–32.3%) and over a third (42%) had a heaping index >10% (data not shown).

Most of the mothers enrolled in the studies had primary and lower secondary educations (median by study: 68.5%, IQR 47.6%–78.7%), a third were between 20 and 24 years of age (median: 33.0%, IQR 29.1%–39.8%) and a third had no previous births (median: 29.0%, IQR 21.2%–40.6%) (Table 2). Most deliveries took place at a health facility, but this varied by study (median: 70.0%, IQR 43.3%–88.0%). Almost all babies were delivered vaginally (median: 94.2%, IQR 92.5%–97.1%) and were singletons (98.0%, IQR 97.2%–98.5%) (Table 2, Table S4 by study). Median female sex of the infants was 48.9% (IQR 48.2%–49.9%) and no intersex babies were reported in the studies.

**TABLE 2:** Demographic characteristics of the included studies, median and interquartile range (IQR) of the included studies.

	Median, % (Interquartile range, %)
Years of education of mother	
No formal education (0 years)	25.7 (8.0–40.8)
Primary and lower secondary (≤11 years)	68.5 (47.6–78.7)
Upper secondary and above (≥12 years)	4.5 (2.7–11.7)
Missing	0.2 (0–0.4)
Age of mother	
<15 years	0.1 (0–0.5)
15–19 years	16.5 (11.3–24.7)
20–24 years	33.0 (29.1–39.8)
25–29 years	24.9 (21.6–27)
30–39 years	20 (11.7–25.4)
≥40 years	1.4 (0.5–2.2)
Missing	0.3 (0–0.5)
Place of delivery	
Outside of facility	27.9 (11.8–53.1)
At facility	70.0 (43.3–88.0)
Missing	0.5 (0–1.8.0)
Type of delivery	
Vaginal	94.2 (92.5–97.1)
Caesarean	5.7 (1.7–7.5)
Missing	0.8 (0–1.2)
Parity	
0	29.0 (21.2–40.6)
1	26.6 (23.1–30.5)
2	17.7 (15.4–19.1)
3	11.6 (7.3–13.1)
≥4	11.5 (4.5–20.4)
Missing	0.1 (0–0.6)
Number born	
Singleton	98.0 (97.2–98.5)
Multiples	2.1 (1.6–2.8)
Infant gender	
Male	51.1 (50.1–51.8)
Female	48.9 (48.2–49.9)

The missingness of newborn type was primarily driven by missing birthweight, especially among the neonatal deaths (Table S5). In Tanzania study 2, Tanzania study 3 and India study 1, we were unable to perform the imputation due to data access/availability, but more than 90% of birthweights were measured in the first 24 hours after delivery and birthweight missingness was very low. China, Brazil, Burkina Faso, Tanzania study 1 and Zambia study 2 had higher missingness (ranging from 37.8% to 12.2% among the neonatal deaths) and more than 90% of birthweights were measured in the first 24 hours. Our recalibration protocol does not improve on weights measured in the first 24 hours after delivery, so we did not perform the recalibration for these studies and instead used the measured weights to conduct the multiple imputation for the missing weights.

For the other studies, we recalibrated the birthweights to time of delivery and used those for the multiple imputation. In our recalibration model, twin/triplets, first‐born infants and babies that later died during the neonatal period had a lower estimated birthweight. Higher gestational age at delivery, higher maternal age and educational status, and male sex were associated with increased estimated birthweight (Table S6). We imputed a birthweight for 11 301/246 336 surviving neonates (4.6%) and 2815/6636 (42.4%) of the neonatal deaths. After birthweight imputation, 5.2% of the deaths and 5.9% of the surviving infants were excluded due to missing or improbable data, resulting in 238 203 live births of a known type.

**FIGURE 2:**
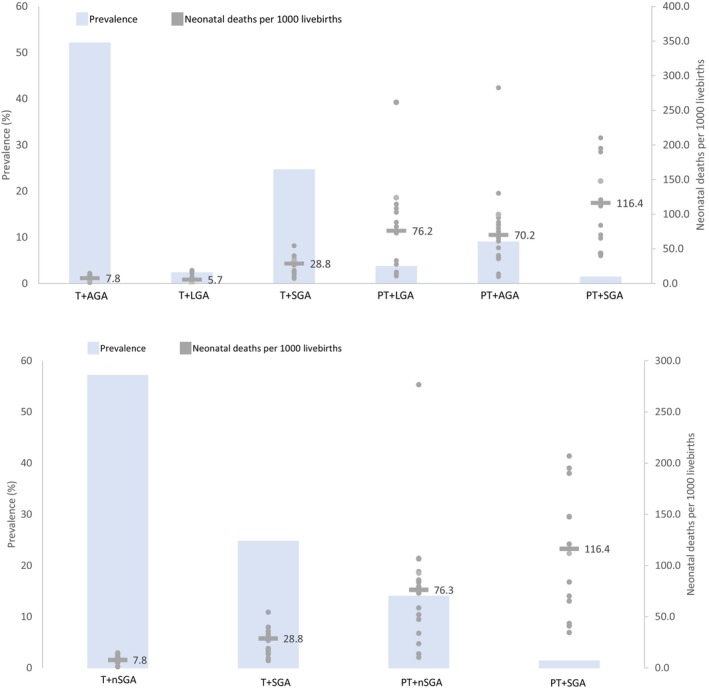
Median prevalence and neonatal mortality risk by study, four‐ and six‐type categorisation. AGA, appropriate for gestational age; LGA, large for gestational age; PT, preterm; SGA, small for gestational age; T, term.

In these studies, T + AGA babies were the most prevalent (median: 52.1%, IQR 40.5%–61.6%), followed by T + SGA (median: 24.7%, IQR 18.8%–41.5%) and then PT + AGA (median: 9.3%, IQR 7.8%–11.6%) (Figure 2, Table S7 by study). PT + LGA, T + LGA and PT + SGA had median prevalences of <5%. T + AGA and T + LGA had similar neonatal mortality rates (median 7.8 deaths per 1000 livebirths, IQR 6.5–13.0 and median 5.7, IQR 0–9.4, respectively). T + SGA had the next highest mortality rate (median 28.8, IQR 16.7–30.5), followed by PT + AGA and PT + LGA (median 70.2, IQR 37.3–90.1 and median 76.2, IQR 22.1–105.6, respectively). PT + SGA had the highest median mortality rate (median: 116.4, IQR 66.5–147.8). The collapsed T + AGA/LGA category (T + nonSGA) had a median of 57.2% by study (IQR 41.8%–65.3%) and neonatal mortality rate of 7.8 (IQR 6.4–13.1). The PT + nonSGA median prevalence was 14.2% (IQR 13.2%–17.6%) and the median mortality rate was 76.3 (IQR 37.9–92.9).

Compared with T + AGA babies, T + LGA had similar risk of death (median RR 0.9, IQR 0–1.0) (Table 3, Table S7 by study). All other types had a higher risk of deaths: the risk of T + SGA babies dying in the neonatal period was 2.6 times higher (IQR 2.0–2.9), the risk of PT + LGA and PT + AGA babies dying was approximately seven times higher (median RR 7.3, IQR 2.3–10.4 and median RR 6.0, IQR 4.4–13.2, respectively) and the risk of PT + SGA babies dying was over 10 times higher (median RR 10.4, IQR 8.6–13.9). Compared with T + nonSGA babies, the median RR for PT + nonSGA babies was 6.0 (IQR 4.1–14.5) (Table 3, Table S8 by study).

**TABLE 3:** Relative risk of neonatal mortality for the six types (reference: Term + AGA), four types (reference: Term + nonSGA) and 10 types (reference: Term + AGA + nonLBW), median and interquartile range (IQR) of the studies.

	Relative risk of neonatal mortality
	Median (Interquartile range); number of studies with sufficient data on type
Six newborn types	
T + AGA	Reference
T + LGA	0.9 (0–1.0); *n* = 16
T + SGA	2.6 (2.0–2.9); *n* = 16
PT + LGA	7.3 (2.3–10.4); *n* = 15
PT + AGA	6.0 (4.4–13.2); *n* = 16
PT + SGA	10.4 (8.6–13.9); *n* = 14
Four newborn types	
T + nonSGA	Reference
T + SGA	2.7 (2.0–3.2); *n* = 16
PT + nonSGA	6.0 (4.1–14.5); *n* = 16
PT + SGA	10.4 (8.5–14.5); *n* = 14
Ten newborn types	
T + AGA + nonLBW	Reference
T + AGA + LBW	1.8 (0.2–3.0); *n* = 10
T + LGA + nonLBW	0.8 (0–0.9); *n* = 16
T + SGA + nonLBW	1.7 (1.4–2.2); *n* = 16
T + SGA + LBW	4.3 (2.8–5.7); *n* = 16
PT + LGA + nonLBW	1.1 (0.8–2.1); *n* = 15
PT + LGA + LBW	23.1 (16.2–40.6); *n* = 14
PT + AGA + nonLBW	1.6 (1.4–1.9); *n* = 15
PT + AGA + LBW	13.0 (8.9–26.4); *n* = 16
PT + SGA + LBW	10.6 (8.8–14.7); *n* = 14

Abbreviations: AGA, appropriate for gestational age; LGA, large for gestational age; nonSGA, non‐SGA (AGA and LGA combined); PT, preterm; SGA, small for gestational age; T, term.

Among the T + SGA, the median RR for babies who were also LBW was 4.3 (IQR 2.8–5.7) and the median RR was 1.7 (IQR 1.4–2.2) for babies who were not LBW. PT + LGA + LBW had a much higher median RR (23.1, IQR 16.2–40.6) compared with PT + LGA + nonLBW (RR 1.1, IQR 0.8–2.1). Finally, PT + AGA babies who were LBW also had a higher median RR (13.0, IQR 8.9–26.4) compared with babies born non‐LBW (RR 1.6, IQR 1.4–1.9) (Table 3, Table S9 by study).

## 4 DISCUSSION

We identified 16 subnational datasets from nine low‐ and middle‐income countries with data collection from 2000 to 2017 to estimate the neonatal mortality risk of vulnerable newborn types. This analysis provides information on newborn health in geographical settings where neonatal mortality is the highest globally but data availability is the lowest.

Newborns born at term or later and SGA, and preterm babies born either LGA or AGA had elevated mortality risk. Preterm newborns born SGA had the highest risk; they were 10 times more likely to die in the first month but had the lowest prevalence (1%). Of all the vulnerable newborns with increased mortality, the highest prevalence was for T + SGA (25%), with 2.5 times the risk of mortality compared with T + AGA babies. We found no additional risk of LGA in this sample. For preterm babies, AGA and LGA mortality rates and relative risks were similar and T + LGA babies had equivalent mortality risks to T + AGA babies. Generally, the RR for each type is lower than the national data from higher income countries.^9^ This is due to the higher mortality risk of babies in the reference group (T + AGA, 7.8 deaths per 1000 livebirths) compared with the national datasets in high income countries (T + AGA, 0.6 deaths per 1000 live births); this difference has been documented in other studies on preterm and SGA mortality.^4^

There is considerable variation in our estimates by study, related to the heterogeneity of the underlying populations. These studies represent geographical variation in LMICs, but temporal variation as well. For instance, we have three studies in different regions of Tanzania; the 2001–2004 study in urban Dar es Salaam had a neonatal mortality rate of 28.5 deaths per 1000 livebirths and the 2012–2013 study in Dar es Salaam and Morogoro regions had 9.5 deaths per 1000 livebirths.

This sub‐sample of studies that collected mortality data was similar to the set of studies used for birth type prevalence estimates, but there is a slightly higher proportion of vulnerable newborns.^9^ In the studies for the prevalence estimates, 58.5% were T + AGA versus 52.1% in the mortality sub‐sample and 21.9% T + SGA versus 24.7% in the mortality sample. The other vulnerable type prevalences were similar: 7.4% versus 9.3% for PT‐AGA, 3.3% versus 2.4% for T + LGA, 1.7% versus 3.7% for PT + LGA, and PT + SGA was the same as the mortality sample. The higher proportion of SGA is likely due to the study site locations; in this analysis almost half of the studies were in Southern Asia, which has the highest regional prevalence of SGA.^42^

We chose commonly used categorical definitions for preterm, SGA and LGA to define vulnerable newborns. The 10th centile definition for SGA has been used since the 1960s but further studies are needed to determine whether these definitions should be revised.^43^ Additionally, there is evidence that revising the definition of LGA as >97th centile would better discriminate the vulnerable babies.^44^ We also recognise the importance of capturing the risk of babies born extremely early or post‐term, but for simplicity in this initial examination of vulnerable newborn type risk, we restricted ourselves to term and preterm. Finally, we used the INTERGROWTH‐21st international standard allowing for direct comparison across many studies.

The four‐ or six‐type definitions are less complex and easier to interpret for programme and policy improvements compared with the ten‐type definition; however, there is evidence that the lower birthweights within preterm and SGA types confer higher mortality risk. T + SGA, PT + LGA and PT + AGA babies who were also LBW had higher relative risk compared with their non‐LBW counterparts, a finding reported in other analyses (reference: T + AGA + nonLBW). In an analysis from the CHERG study, babies born T + SGA + nonLBW had a RR of 1.89 of neonatal mortality, compared with 4.77 for T + SGA babies born LBW.^45^ Birthweights provide additional information on mortality risk for each of the vulnerable types, even if just indicating babies born at the lower centile of SGA. PT + LGA + LBW babies also had additional mortality risk compared with their nonLBW counterparts, but this is likely a measurement artefact. To be considered PT, LGA and LBW, boys must be born <33 weeks and girls born <33^+4^ weeks gestational age, so the mortality risk is likely associated with being born early, rather than LGA.

In addition to the limitations presented in the subnational prevalence paper in this series, the main limitation of this mortality analysis is the missing birthweights, especially among neonatal deaths.^9^ We imputed almost half of the neonatal deaths used in this analysis (42%, Figure 1). Many of our studies were community‐based (*n* = 11 studies) and, for the home deliveries, early neonatal deaths occurred before the study team could arrive at the home to weigh the baby (Table S3). Additionally, newborns typically lose weight in the first 2–3 days of life due to fluid losses until the establishment of breastfeeding. Weight measured in the 2–3 days after delivery, at the nadir of early neonatal weight loss, inflates estimates of SGA and underestimates LGA.^12^, ^46^ Using only the measured birthweight to calculate mortality risk by type would have underestimated the overall mortality rates and the mortality risk of certain vulnerable newborn types.

We aimed to address this bias using the recalibrated birthweights to generate imputed birthweight. The recalibrated weights were based on a longitudinal sample of singleton newborns in rural Nepal born between 2002 and 2005 who survived at least 10 days. There is evidence that babies may have different early neonatal growth patterns in different regions due to underlying population health or newborn feeding practices. A study in Tanzania of early neonatal weight change found an earlier nadir (27 hours for boys and 28 hours for girls) than that measured in the Nepal study (2.1 days), although the mean weight loss at the nadir was similar (4.7% in Tanzania and 4.3% in Nepal).^12^, ^47^ A study cohort of infants from Nepal, Pakistan, Guinea‐Bissau and Uganda found a similar median nadir of two days, with an average mean weight loss of 5.9%, and babies born LBW had a slower growth trajectory over 30 days.^48^ Also, to be included in the rural Nepal longitudinal sample of weights measured in the first 10 days, the baby must have survived the early neonatal period. We included a covariate adjusting for neonatal death for the multiple imputations (Table S6) but we do not have any information on how well our recalibration model estimates weight at time of delivery for early neonatal deaths.

For a subset of the studies, we compared the median birthweight, and four‐ and six‐type neonatal mortality rates and relative risks using (1) birthweights measured <72 hours after delivery with missing birthweight excluded, and (2) birthweights using the recalibration and/or imputation method (Tables S10–S12).

In the studies where only imputation (not recalibration) was used, the median birthweight using the imputation did not change or was <5 g different (China, Brazil and Zambia study 2) or increased (Burkina Faso). In studies where the recalibration with imputation was used, the median birthweight increased for the Bangladesh study 3 (+50 g), India study 1 (+40 g), Nepal study 1 (+40 g), Nepal study 2 (+21 g) and Zambia study 1 (78 g) studies, with the exception of Malawi, where it essentially stayed the same.

The recalibration protocol estimates a weight closer to the time of birth, and many of these studies had a significant portion of babies measured at the nadir of early neonatal weight loss (estimated 1–2 days after delivery). Therefore, for these five studies, the median birthweight was increased slightly because an estimated birthweight (at time of delivery) was higher than that measured, as many of the infants were measured at the nadir.

As expected, the mortality risks of all types increased when using the recalibration and/or imputation method, as a birthweight is now imputed for early neonatal deaths, where previously they were excluded (Tables S11 and S12). In the four‐type categorisation, the median RR of T + SGA did not change and the median RR of nonSGA + PT increased from 4.5 to 5.9 (Table S11). The median RR of SGA + PT decreased from 13.4 to 10.4. For the six‐type categorisation, the median RR for T + LGA, T + SGA and PT + AGA were similar using the measured and imputed birthweights (1.0 versus 0.9; 2.1 versus 2.0 and 5.7 versus 5.9, respectively). The median RR of PT + LGA increased from 2.2 to 7.6 and the median RR of PT + SGA decreased from 12.1 to 10.0 (Table S12).

Our method estimated more neonatal deaths with a missing birthweight as PT + LGA using the six‐type categorisation (PT + nonSGA for the four‐type) and fewer for PT + SGA. This could be due to an actual biological construct, measurement error with gestational age or our model overestimating birthweights for preterm babies. However, we consider this model an improvement on the measured birthweight data given we can include the neonatal deaths with missing birthweight, critical for this analysis on neonatal mortality risk. Most studies used LMP collected during pregnancy (*n* = 12) to calculate gestational age. There could be measurement error that impacted the size‐for‐gestational age estimates. Although ultrasound measurement in the <24‐week period is recommended by WHO for ascertainment of gestational age, LMP is adequate in areas where access to ultrasound is limited.^49^, ^50^, ^51^ We also used birthweight standard curves, instead of fetal weight standard curves, which underestimate FGR for preterm babies, as the pathology that leads to FGR may also induce preterm births.^52^ However, there are also limitations with use of a universal fetal growth standard in international settings. A study applying three different fetal growth standards found important differences in classification of SGA and LGA babies, indicating more work is needed on universal standards of fetal growth.^53^

A final limitation was that we presented crude measures of mortality risk for newborn types. Potential confounders of neonatal mortality risk and newborn type range by socio‐economic factors, underlying health of the maternal population, health system factors and many more exposures. There was limited information on spontaneous versus vacuum‐ or forceps‐assisted vaginal delivery, emergency versus planned caesarean section, and presentation of the newborn (i.e. breech). We were limited by data collected in the studies and hope to address this in future research.

This analysis is possible due to the generous collaboration of our co‐authors and represents what is achievable with increased data availability and sharing. As health data systems improve in completeness and quality, countries will be able directly to track the health of vulnerable newborns but, until then, the global health community relies on research data. The authors support continued openness and availability of de‐identified, individual‐level study data.

Babies in low‐ and middle‐income settings who are preterm or growth‐restricted have considerable mortality risk compared with full term and not growth‐restricted babies born in the same location. All preterm types had higher neonatal mortality risks compared with the term types and there was compounding risk of preterm with SGA. Term SGA babies have lower risk compared with preterm babies but are the most prevalent vulnerable newborn type. Four‐ or six‐type definitions were less complex to calculate and interpret, especially the four‐type definition, as we did not find evidence of differential risk between AGA and LGA babies in this sample. The ten‐type definition shows that babies with LBW have higher risks but, as an population‐level indicator of neonatal health, this is difficult to calculate and interpret, and some categories are measurement artefacts such as the PT + LGA + LBW, which only captures early preterm babies (<33 weeks for boys and <33^+4^ weeks for girls), likely indicating the risk of early preterm rather than LGA or LBW status.

This study provides critical information on vulnerable newborn health in areas where the burden is the highest but data availability is the lowest. The classification of births as preterm and/or SGA may assist in the understanding of the social determinants and biomedical risk factors that are important to design and implement preventive interventions, as well as improved management of vulnerable newborns.

## AUTHOR CONTRIBUTIONS

The Vulnerable Newborn Measurement Collaboration was planned by JEL and REB. This analysis was designed by DE, EH, JK and ACL with REB. All authors contributed to the study protocol and analysis. Descriptive analysis of the datasets was undertaken by DE, EH, MD and LSW. The paper was drafted by EH with DE, JK, ACL, MD, and REB. All authors helped revise the paper. All authors reviewed and agreed on the final version.

## FUNDING INFORMATION

The Children's Investment Fund Foundation, grant 2004‐04670. The funders had no role in the study design, data collection, analysis or interpretation of the paper.

## CONFLICT OF INTEREST STATEMENT

None declared.

## ETHICS APPROVAL

The Vulnerable Newborn Measurement Collaboration was granted ethical approval by the Institutional Review Boards of the London School of Hygiene & Tropical Medicine (ref: 22858) and Johns Hopkins University (ref: 16439). All collaborators received local ethical permission for their data where relevant.

## Supporting information


supportingInformation

## Data Availability

Data sharing and transfer agreements were jointly developed and signed by all collaborating partners. The pooled summary table data generated during the current study have been deposited online with data access subject to approval at https://doi.org/10.17037/DATA.00003095.

## APPENDIX 1

### Subnational Collaborative Group for Vulnerable Newborn Mortality*

Rolf D. W. Klemm, Allan B. Massie, Maithilee Mitra, Sucheta Mehra, Kerry J. Schulze, Alfred Sommer, Md. Barkat Ullah, Alain B. Labrique, Mabhubur Rashid, Saijuddin Shaikh, Nazma Begum, Nabidul Haque Chowdhury, Md. Shafiqul Islam, Rasheda Khanam, Dipak Kumar Mitra, Abdul Quaiyum, Modiegi Diseko, Joseph Makhema, Yue Cheng, Meselech Roro, Bilal Shikur Endris, Charles D. Arnold, Rajiv Bahl, Nita Bhandari, Jose Martines, Sarmila Mazumder, Lotta Hallamaa, Juha Pyykkö, Willy Urassa, Phillippe Deloron, Ib Christian Bygbjerg, Sofie Lykke Moeller, Thor Grundtvig Theander, Alfa Muhihi, Ramadhani Abdallah Noor, Moses R. Kamya, Miriam Nakalembe, Godfrey Biemba, Julie M. Herlihy, Reuben K. Mbewe, Fern Mweena, Kojo Yeboah‐Antwi, Andrew Prendergast.

### Vulnerable Newborn Measurement Core Group

**LSHTM:** Joy E. Lawn; Hannah Blencowe; Eric Ohuma; Yemi Okwaraji; Judith Yargawa; Ellen Bradley; Lorena Suarez Idueta

*Individuals involved in multiple studies on this list are only named once.
