# Supplementary material for: Neonatal mortality risk of vulnerable newborns: A descriptive analysis of subnational, population‐based birth cohorts for 238 203 live births in low‐ and middle‐income settings from 2000 to 2017
Source: BJOG. 2023 May 8;132(Suppl 8):S48–59. doi: 10.1111/1471-0528.17518 (PMC12678067; doi:10.1111/1471-0528.17518)
Supplement: Supplementary file 1 — supportingInformation [file BJO-132-S48-s001.docx]

**Supplementary**

**Table S1: Strobe checklist**(1)

|  | Item No | Recommendation | Page  No. |
| --- | --- | --- | --- |
| **Title and abstract** | 1 | (*a*) Indicate the study’s design with a commonly used term in the title or the abstract | 1 |
|  |  | (*b*) Provide in the abstract an informative and balanced summary of what was done and what was found | 5 |
| Introduction | | |  |
| Background/rationale | 2 | Explain the scientific background and rationale for the investigation being reported | 6 |
| Objectives | 3 | State specific objectives, including any prespecified hypotheses | 6 |
| Methods | | |  |
| Study design | 4 | Present key elements of study design early in the paper | 6 |
| Setting | 5 | Describe the setting, locations, and relevant dates, including periods of recruitment, exposure, follow-up, and data collection | 6,7 |
| Participants | 6 | (*a*) *Cohort study*—Give the eligibility criteria, and the sources and methods of selection of participants. Describe methods of follow-up  *Case-control study*—Give the eligibility criteria, and the sources and methods of case ascertainment and control selection. Give the rationale for the choice of cases and controls  *Cross-sectional study*—Give the eligibility criteria, and the sources and methods of selection of participants | 6,7 |
|  |  | (*b*) *Cohort study*—For matched studies, give matching criteria and number of exposed and unexposed  *Case-control study*—For matched studies, give matching criteria and the number of controls per case |  |
| Variables | 7 | Clearly define all outcomes, exposures, predictors, potential confounders, and effect modifiers. Give diagnostic criteria, if applicable | 7,8 |
| Data sources/ measurement | 8 | For each variable of interest, give sources of data and details of methods of assessment (measurement). Describe comparability of assessment methods if there is more than one group | 7,8 |
| Bias | 9 | Describe any efforts to address potential sources of bias | 11,12 |
| Study size | 10 | Explain how the study size was arrived at | Not applicable |
| Quantitative variables | 11 | Explain how quantitative variables were handled in the analyses. If applicable, describe which groupings were chosen and why | 7,8 |
| Statistical methods | 12 | (*a*) Describe all statistical methods, including those used to control for confounding | 8 |
|  |  | (*b*) Describe any methods used to examine subgroups and interactions | 8 |
|  |  | (*c*) Explain how missing data were addressed | 7 |
|  |  | (*d*) *Cohort study*—If applicable, explain how loss to follow-up was addressed  *Case-control study*—If applicable, explain how matching of cases and controls was addressed  *Cross-sectional study*—If applicable, describe analytical methods taking account of sampling strategy | Not applicable |
|  |  | (*e*) Describe any sensitivity analyses |  |
| Results | | | |
| Participants | 13 | (a) Report numbers of individuals at each stage of study—eg numbers potentially eligible, examined for eligibility, confirmed eligible, included in the study, completing follow-up, and analysed | 8 |
|  |  | (b) Give reasons for non-participation at each stage | 8 |
|  |  | (c) Consider use of a flow diagram | 8 |
| Descriptive data | 14 | (a) Give characteristics of study participants (eg demographic, clinical, social) and information on exposures and potential confounders | 8,9 |
|  |  | (b) Indicate number of participants with missing data for each variable of interest | 9 |
|  |  | (c) *Cohort study*—Summarise follow-up time (eg, average and total amount) |  |
| Outcome data | 15 | *Cohort study*—Report numbers of outcome events or summary measures over time | 9,10 |
|  |  | *Case-control study—*Report numbers in each exposure category, or summary measures of exposure |  |
|  |  | *Cross-sectional study—*Report numbers of outcome events or summary measures |  |
| Main results | 16 | (*a*) Give unadjusted estimates and, if applicable, confounder-adjusted estimates and their precision (eg, 95% confidence interval). Make clear which confounders were adjusted for and why they were included | Not applicable |
|  |  | (*b*) Report category boundaries when continuous variables were categorized | 7,8 |
|  |  | (*c*) If relevant, consider translating estimates of relative risk into absolute risk for a meaningful time period | Not applicable |
| Other analyses | 17 | Report other analyses done—eg analyses of subgroups and interactions, and sensitivity analyses | 8,9  11,12 |
| Discussion | | |  |
| Key results | 18 | Summarise key results with reference to study objectives | 10 |
| Limitations | 19 | Discuss limitations of the study, taking into account sources of potential bias or imprecision. Discuss both direction and magnitude of any potential bias | 11,12 |
| Interpretation | 20 | Give a cautious overall interpretation of results considering objectives, limitations, multiplicity of analyses, results from similar studies, and other relevant evidence | 12,13 |
| Generalisability | 21 | Discuss the generalisability (external validity) of the study results | 11,12 |
| Other information | | |  |
| Funding | 22 | Give the source of funding and the role of the funders for the present study and, if applicable, for the original study on which the present article is based | 14 |

**Table S2: Description of studies**

| **Country** | **Period of**  **observation** | **GA type** | **Total**  **LBs** | **Total NN deaths** | **Mortality rate per 1000 LB**^[[1]](#footnote-1)^ | **LTFU** | **Weight measured <24 hours**^[[2]](#footnote-2)^ **(%)** | **Missing phenotype surviving**  **% (n/N)** ^[[3]](#footnote-3)^ | **Missing phenotype deaths**  **% (n/N)** | **Birthweight adjustment method** |
| --- | --- | --- | --- | --- | --- | --- | --- | --- | --- | --- |
| **Eastern Asia** | | | | | | | | | | |
| China(2) | 2002-2006 | LMP/s | 4,697 | 73 | 15.5 | 0 | 96.6 | 6.5  (299/4624) | 21.9  (16/73) | Imputation of missing weights |
| **Latin America** | | | | | | | | | | |
| Brazil (3) | 2015 | LMP/US | 4,275 | 37 | 8.7 | 0 | 100 | 0.1  (4/4238) | 37.8  (14/37) | Imputation of missing weights |
| **sub-Saharan Africa** | | | | | | | | | | |
| Burkina Faso(4,5) | 2004-2006  2006-2008 | LMP/US | 2,562 | 49 | 20.3 | 147 | 100 | 17.5  (413/2366) | 12.2  (6/49) | Imputation of missing weights |
| Malawi(6,7) | 2003-2006  2011-2012 | US | 2,540 | 71 | 28.3 | 33 | 50.5 | 9.8  (216/2436) | 54.9  (39/71) | Recalibration of all weights & imputation of missing |
| Tanzania 1 (8) | 2001 – 2004 | LMP/s | 8,137 | 215 | 27.4 | 287 | 100 | 4.9  (369/7537) | 8.4  (18/215) | Imputation of missing weights |
| Tanzania 2 (9,10) | 2008-2010  2014-2016 | US | 1,445 | 37 | 29.9 | 104 | 97.0 | 8.4  (101/1200) | 5.4  (2/37) | Exclude weights measured >72 hours |
| Tanzania 3 (11) | 2012-2013^[[4]](#footnote-4)^ | LMP/s & LMP/d | 10,893 | 101 | 9.9 | 72 | 70.3 | 23.8  (2568/10720) | 15.5^[[5]](#footnote-5)^  (16/101) | Exclude weights measured >72 hours |
| Zambia 1 (12) | 2011 - 2013 | LMP/s | 37,856 | 528 | 14.4 | 1156 | 62.0 | 20.9  (7547/36172) | 65.0  (343/528) | Recalibration of all weights & imputation of missing weights |
| Zambia 2 (13) | 2015 – 2017 | LMP/s | 11,016 | 135 | 12.3 | 0 | 95.0 | 13.6  (1477/10881) | 20.7  (28/135) | Imputation of missing weights |
| **Southern Asia** | | | | | | | | | | |
| Bangladesh 1 (14) | 2001-2007 | LMP/s | 18,176 | 786 | 43.2 | 0 | 61.9 | 2.81  (489/17390) | 3.05  (24/786) | Recalibration of all weights & imputation of missing weights |
| Bangladesh 2 (15) | 2007-2012 | LMP/s | 28,516 | 1246 | 43.7 | 0 | 67.8 | 4.94  (1347/27270) | 9.15  (114/1246) | Recalibration of all weights & imputation of missing weights |
| Bangladesh 3  (16,17) | 2011-2014 | LMP/s | 21,227 | 918 | 45.1 | 874 | 75.9 | 13.3  (2576/19435) | 51.3  (471/918) | Recalibration of all weights & imputation of missing weights |
| India 1 (18) | 2000-2001 | LMP/s | 5,890 | 196 | 33.3 | 7 | 37.5 | 28.7  (1632/5687) | 60.2  (118/196) | Recalibration of all weights & imputation of missing weights |
| India 2 (19) | 2010-2012 | LMP/s | 44,984 | 579 | 12.9 | 0 | 64.9 | 0.1  (26/44405) | 0  (0/579) | Exclude measured weights >72 hours |
| Nepal 1(20,21) | 2002-2005 | LMP/s | 23,665 | 708 | 30.0 | 88 | 88.2 | 8.1  (1862/22869) | 48.9  (346/708) | Recalibration of all weights & imputation of missing weights |
| Nepal 2(22) | 2010-2017 | LMP/s | 31,116 | 957 | 31.9 | 1,157 | 66.0 | 22.6  (6566/29002) | 61.8  (591/957) | Recalibration of all weights & imputation of missing weights |

LB: livebirths; NN: neonatal; LTFU: lost-to-follow-up; LMP/s: Last menstrual period (LMP) collected during pregnancy surveillance; LMP/d: Last menstrual period (LMP) collected at delivery; LMP/US: combination of ultrasound (US) and LMP

**Table S3: Characteristics of the studies included in this analysis**

| **Study ID** | **Setting** | **Study Design** | **Population represented** |
| --- | --- | --- | --- |
| **Eastern Asia** |  |  |  |
| China | Rural Shaanxi Province | Cluster RCT of antenatal supplementation with micronutrients interventions | Population based recruitment of all pregnant women in study area |
| **Latin America** |  |  |  |
| Brazil | Urban Pelotas City | Prospective observational cohort study | Longitudinal birth cohort of all children delivered in the 5 maternity hospitals in the city of Pelotas |
| **sub-Saharan Africa** |  |  |  |
| Burkina Faso | Rural Hounde | - RCT of multiple micronutrient supplementation - RCT of maternal fortified food supplementation | Prospective, community-based cohort |
| Malawi | Rural Mangochi District | - RCT of sulfadoxine-pyrimethamine and azithromycin in pregnancy - RCT of lipid-based nutrient supplement | Facility-based ANC clinic recruitment of pregnancies |
| Tanzania 1 | Urban Dar es Salaam | RCT of multiple micronutrient supplementation | Facility-based ANC clinic recruitment of pregnancies |
| Tanzania 2 | Rural Korogwe | Prospective observational cohort studies | - Population-based recruitment of women preconception or in the 1st trimester of pregnancy - Facility-based ANC clinic recruitment of pregnancies |
| Tanzania 3 | Morogoro and Dar es Salaam regions | RCT of newborn Vitamin A supplementation | Facility-based ANC clinic and labour ward recruitment of pregnancies |
| Zambia 1 | Urban and rural areas of Southern Province | RCT of chlorhexidine application for umbilical cord disinfection | Facility-based ANC clinic recruitment of pregnancies |
| Zambia 2 | Urban Lusaka | Prospective observational cohort study | Population based recruitment of all pregnant women in study area |
| **Southern Asia** |  |  |  |
| Bangladesh 1 | Rural Gaibandha and Rangpur | Cluster RCT of maternal Vitamin A supplementation | Population-based recruitment of all pregnant women in study area |
| Bangladesh 2 | Rural Gaibandha and Rangpur | Cluster RCT of antenatal multiple micronutrient | Population-based recruitment of all pregnant women in study area |
| Bangladesh 3 | Rural Sylhet | Prospective observational cohort study | Population-based recruitment of all pregnant women in study area |
| India 1 | Rural Tamil Nadu | RCT of newborn Vitamin A supplementation | Population-based recruitment of all pregnant women in study area |
| India 2 | Rural Haryana | RCT of newborn Vitamin A supplementation | Population-based recruitment of all pregnant women in study area |
| Nepal 1 | Rural Sarlahi District | Cluster RCT of newborn skin-umbilical cord cleansing with chlorhexidine | Population-based recruitment of all pregnant women in study area |
| Nepal 2 | Rural Sarlahi District | Cluster RCT of newborn massage with sunflower seed oil | Population-based recruitment of all pregnant women in study area |

**Table S4: Demographic characteristics by study among infants with known neonatal survival status and type**

|  | China  % (n) | Brazil  % (n) | Burkina Faso  % (n) | Malawi  % (n) | Tanzania 1  % (n) | Tanzania 2  % (n) | Tanzania 3  % (n) | Zambia 1  % (n) | Zambia 2  % (n) |
| --- | --- | --- | --- | --- | --- | --- | --- | --- | --- |
| **Years of education of mother** |  |  |  |  |  |  |  |  |  |
| No formal education  (0 years) | NC | 0.4  (17) | 84.8  (1831) | 36.4 (905) | 7.2  (568) | 7.4  (91) | 9.5  (793) | 8.5  (2462) | 6.7  (670) |
| Primary and lower secondary  (<=11 years) | NC | 68.5 (2926) | 9.9  (213) | 61.4 (1526) | 87.1  (6864) | 79.6  (975) | 86.1  (7152) | 80.4 (23308) | 77.2 (7697) |
| Upper secondary and above  (>=12 years) | NC | 31.1 (1330) | 2.4  (52) | 2.1  (53) | 5.3  (414) | 12.5  (153) | 3  (247) | 11  (3203) | 16.3 (1634) |
| Missing/Don’t know | NC | NA | 2.9  (62) | NA | 0.5  (36) | 0.5  (6) | 0.2  (16) | 0.1  (16) | NA |
| **Age of mother** |  |  |  |  |  |  |  |  |  |
| <15 years | 0 (0) | 0.6  (27) | 0.1  (3) | 0.1 (3) | 0  (1) | 0.2  (2) | 0  (0) | 0  (4) | 0.4  (4) |
| 15-19 years | 8.2  (386) | 13.9  (596) | 24.7  (534) | 22.9 (570) | 7.9  (625) | 11.8  (145) | 18.1  (1504) | 23.3 (6752) | 14.9  (1492_ |
| 20-24 years | 47.5 (2229) | 23.6 (1008) | 31.1  (671) | 28.7 (712) | 39.9  (3143) | 25.6  (314) | 29.6  (2461) | 27.3 (7904) | 34.6  (3459) |
| 25-29 years | 28.1 (1318) | 23.5  (1006) | 22.3  (482) | 25.8 (641) | 30.3  (2385) | 26.9  (330) | 25.0  (2079) | 20.6 (5963) | 24.9  (2494) |
| 30-39 years | 15.9 (746) | 35.3 (1508) | 19.5  (420) | 20.5 (510) | 20.4  (1605) | 17.8  (218) | 25.3  (2099) | 25.5 (7382) | 23.7  (2366) |
| ≥40 years | 0.3  (13) | 3.0  (128) | 1.8  (38) | 1.9  (48) | 1.1  (83) | 14.5  (178) | 2  (166) | 3.4  (984) | 1.7  (172) |
| Missing/Don’t know | NA | NA | 0.5  (10) | NA | 0.5  (40) | 3.1  (38) | 0  (0) | NA | 0.14  (14) |
| **Place of delivery** |  |  |  |  |  |  |  |  |  |
| Outside of facility | 16.5 (775) | 0  (0) | 12.3  (266) | 24.8 (617) | 1.8  (143) | 9.8  (120) | 14.1  (1175) | 31  (8996) | 4.3  (430) |
| At facility | 83.5 (3917) | 100 (4274) | 87.6  (1891) | 73  (1813) | 96.6 (7,617) | 89.6  (1098) | 85.5  (7106) | 66.2 (19179) | 95.4 (9543) |
| Missing/Don’t know | 0  (0) | 0  (0) | 0  (1) | 2.2  (54) | 1.6  (122) | 0.6  (7) | 0.3  (28) | 2.8  (814) | 0.3  (28) |
| **Type of delivery** |  |  |  |  |  |  |  |  |  |
| Vaginal | 90.4 (4240) | NC | NC | 94.1 (2338) | 92.2  (7267) | 90.4  (1107) | 92.5  (7689) | 97.2 (28176) | 97.6 (9761) |
| Caesarean | 8.8  (413) | NC | NC | 4.7  (117) | 7.8  (615) | 9.6  (118) | 7.5  (620) | 1.2  (352) | 1.6  (164) |
| Missing/Don’t know | 0.8  (39) | NC | NC | 1.2  (29) | 0  (0) | 0  (0) | 0  (0) | 1.6  (461) | 0.8  (76) |
| **Parity** |  |  |  |  |  |  |  |  |  |
| No previous live or stillbirths | 64.7 (3038) | 49.4 (2111) | 19.5  (420) | 21.5 (533) | 44.3  (3491) | 20.1  (246) | 14.6  (1216) | 23.8 (6892) | 29.8 (2982) |
| 1 | 31.2 (1466) | 30.9 (1321) | 20.4  (440) | 19.4 (483) | 27.9  (2201) | 24.3  (298) | 30.5  (2534) | 18.5 (5372) | 25.8 (2576) |
| 2 | 3.4  (161) | 11.0  (472) | 15  (324) | 18  (448) | 14.9  (1173) | 21.2  (260) | 22.6  (1880) | 15.6 (4509) | 27.0  (2703) |
| 3 | 0.5  (24) | 4.7  (202) | 12.9  (279) | 16.3 (405) | 7  (548) | 34.4  (421) | 14.5  (1204) | 12.9 (3741) | 13.2 (1320) |
| ≥4 | 0.1  (3) | 3.9  (168) | 32.2  (695) | 24.8 (615) | 5.4  (425) | 20.1  (246) | 16.6  (1376) | 29.2 (8475) | 4.1  (413) |
| Missing/Don’t know | NA | NA | NA | NA | 0.6  (44) | 0  (0) | 1.2  (99) | NA | 0.1  (7) |
| **Number born** |  |  |  |  |  |  |  |  |  |
| Singleton | 98  (4599) | 97.4 (4163) | 96.7  (2086) | 98.7 (2452) | 96.3 (7,592) | 95.4  (1082) | 96.6  (8027) | 97.7 (28319) | 97.6 (9763) |
| Multiples | 2  (93) | 2.6  (111) | 3.3  (72) | 1.3  (32) | 3.7  (290) | 4.6  (52) | 3.4  (282) | 2.3  (670) | 2.3  (232) |
| **Infant sex** |  |  |  |  |  |  |  |  |  |
| Male | 55.2 (2591) | 50.6 (2164) | 50.2  (1083) | 49.7 (1235) | 51.8 (4,085) | 49.5  (606) | 52.3  (4349) | 49.7 (14416) | 49.6 (4961) |
| Female | 44.8 (2101) | 49.4 (2110) | 49.8  (1075) | 50.3 (1249) | 48.2 (3,797) | 50.5  (619) | 47.7  (3960) | 50.3 (14573) | 50.4 (5040) |

**Table S4 continued, Demographic characteristics by study among infants with known neonatal survival status and type**

|  | Bangladesh 1  % (n) | Bangladesh 2  % (n) | Bangladesh 3  % (n) | India 1  % (n) | India 2  % (n) | Nepal 1  % (n) | Nepal 2  % (n) |
| --- | --- | --- | --- | --- | --- | --- | --- |
| **Years of education of mother** |  |  |  |  |  |  |  |
| No formal education (0 years) | 39.4  (6958) | 25.7  (6950) | 24.4  (4895) | 39.7  (2234) | 41.8 (18,801) | 76.1 (17,762) | 67.3 (20,119) |
| Primary and lower secondary (<=11 years) | 57.5  (10,159) | 70.8  (19,151) | 73.6  (14,803) | 48.3  (2719) | 46.8 (21,039) | 23.1  (5382) | 28.2  (8419) |
| Upper secondary and above (>=12 years) | 3.1  (344) | 3.5  (694) | 2.0  (402) | 12  (673) | 11.4  (5118) | 0.8  (186) | 4.5  (1349) |
| Missing | NA | NA | NA | NA | 0 | NA | NA |
| **Age of mother** |  |  |  |  |  |  |  |
| <15 years | 5  (888) | 1.3  (362) | 0.3  (64) | 0  (1) | 0  (0) | 0.5  (121) | 0.7  (195) |
| 15-19 years | 37.9  (6693) | 32.7  (8846) | 9.9  (1987) | 14.3  (805) | 7.9  (3560) | 24.8  (5782) | 34.4 (10,268) |
| 20-24 years | 29.2  (5166) | 32.6  (8814) | 33.4  (6706) | 49.3  (2772) | 54  (24,298) | 39.8  (9283) | 39.5 (11,808) |
| 25-29 years | 17.3  (3049) | 20.8  (5621) | 24.8  (4988) | 28.3  (1591) | 27.2 (12,226) | 21.8  (5089) | 17.7  (5288) |
| 30-39 years | 10.1  (1779) | 12.1  (3268) | 28.7  (5760) | 8  (449) | 10.3  (4641) | 12.2  (2843) | 7.5  (2230) |
| ≥40 years | 0.5  (88) | 0.5  (144) | 3  (595) | 0.1  (8) | 0.5  (233) | 0.9  (212) | 0.3  (98) |
| Missing | NA | NA | NA | NA | 0 | NA | NA |
| **Place of delivery** |  |  |  |  |  |  |  |
| Outside of facility | 90.8  (16,038) | 82.5  (22,309) | 88.5  (17,796) | 32.6  (1834) | 43.3  (19,508) | 86.2 (20,105) | 37.8 (11,289) |
| At facility | 7.5  (1329) | 16  (4329) | 11.5  (2303) | 67  (3771) | 56.6  (25,450) | 8.7  (2037) | 52.4 (15671) |
| Missing | 1.7  (296) | 1.5  (417) | 0 (1) | 0.4  (21) | 0 | 5.1  (1188) | 9.8  (2927) |
| **Type of delivery** |  |  |  |  |  |  |  |
| Vaginal | 96.6  (17,060) | 93.1  (25,184) | NC | 94.2  (5300) | 94.2  (42,368) | 100 (23,331) | 97.1 (29,022) |
| Cesarean | 1.7  (304) | 5.7  (1542) | NC | 5.8  (326) | 5.8  (2,590) | 0 | 2.9  (865) |
| Missing | 1.7  (299) | 1.2  (329) | NC | 0 (0) | 0 | 0 | 0 (0) |
| **Parity** |  |  |  |  |  |  |  |
| No previous live or stillbirths | 46.6  (8235) | 5.6  (1528) | 28.2  (5662) | 39.3  (2211) | 30.2 (13,597) | 26  (6058) | 31.5  (9403) |
| 1 | 23.7  (4190) | 32.5  (8803) | 21.8  (4390) | 30.6  (1720) | 29  (13,027) | 24  (5610) | 27.4  (8181) |
| 2 | 15.5  (2735) | 16.9  (4575) | 17.3  (3474) | 18.5  (1041) | 18.5  (8327) | 18.9  (4421) | 19.7  (5875) |
| 3 | 7.8  (1370) | 7.2  (1938) | 12.3  (2468) | 7.3  (411) | 10.1  (4553) | 13.1  (3060) | 10.8  (3231) |
| ≥4 | 6.4 (  619) | 4.5  (727) | 20.4  (4106) | 4.3  (243) | 12.2  (5454) | 17.9  (4181) | 10.7  (3197) |
| Missing | NA | NA | NA | NA | 0  (0) | NA | NA |
| **Number born** |  |  |  |  |  |  |  |
| Singleton | 98.6  (17,408) | 98.3  (26,591) | 97.9  (19,686) | 99  (5568) | 98.7  (44,387) | 98.4 (22,966) | 98.4 (29,403) |
| Multiples | 1.4  (255) | 1.7  (464) | 2.1  (414) | 1  (58) | 1.3  (571) | 1.6  (364) | 1.6  (484) |
| **Infant sex** |  |  |  |  |  |  |  |
| Male | 51  (9006) | 51.4  (13,919) | 51.2  (10,285) | 50.5  (2843) | 52.1  (23,404) | 51.5 (12,021) | 51.8 (15,475) |
| Female | 49  (8657) | 48.6  (13,136) | 48.8  (9815) | 49.5  (2783) | 47.9  (21,554) | 48.5 (11,309) | 48.2 (14,412) |

NA: not applicable - study used recalibration and imputation method to estimate birthweight using this covariate; NC: Data was not collected in the study or was collected by category using different definitions **Table S5: Records excluded by neonatal survival status and study for infants with known survival status, after birthweight imputation**

| **Study ID** | **Missing phenotype, surviving %(n)** | | | | | | **Missing phenotype, neonatal death %(n)** | | | | | | **Total: known type and survival status**^[[6]](#footnote-6)^ |
| --- | --- | --- | --- | --- | --- | --- | --- | --- | --- | --- | --- | --- | --- |
|  | **Total** | **Missing BW**^[[7]](#footnote-7)^ | **Missing GA** | **Impro-bable** | **Missing gender** | **Total excluded**^[[8]](#footnote-8)^ | **Total** | **Missing BW** | **Missing GA** | **Impro-bable** | **Missing gender** | **Total excluded^1^** |  |
| **Eastern Asia** | | | | | | | | | | | | | |
| China | 4,624 | 0 | 0 | 4 | 0 | 5 | 73 | 0 | 0 | 0 | 0 | 0 | 4,692 |
| **Latin America** | | | | | | | | | | | | | |
| Brazil | 4,178 | 0 | 0 | 1 | 0 | 1 | 37 | 0 | 0 | 0 | 0 | 0 | 4,274 |
| **Sub-Saharan Africa** | | | | | | | | | | | | | |
| Burkina Faso | 2,366 | 56 | 60 | 36 | 198 | 253 | 49 | 0 | 0 | 0 | 4 | 4 | 2,158 |
| Malawi | 2,436 | 11 | 0 | 0 | 4 | 11 | 71 | 11 | 0 | 1 | 9 | 12 | 2,484 |
| Tanzania 1 | 7,537 | 0 | 0 | 0 | 22 | 218 | 215 | 0 | 0 | 9 | 1 | 10 | 7,524 |
| Tanzania 2 | 1304 | 109 | 4 | 1 | 13 | 114 | 37 | 2 | 0 | 0 | 0 | 2 | 1,225 |
| Tanzania 3 | 10,720 | 23 | 2251 | 297 | 0 | 2,568 | 101 | 0 | 14 | 2 | 0 | 16 | 8,237 |
| Zambia 1 | 36,172 | 6,205 | 4,384 | 1510 | 332 | 7,604 | 528 | 88 | 74 | 19 | 2 | 107 | 28,989 |
| Zambia 2 | 10,881 | 537 | 441 | 489 | 36 | 1006 | 135 | 8 | 0 | 4 | 0 | 9 | 10001 |
| **Southern Asia** | | | | | | | | | | | | | |
| Bangladesh 1 | 17,390 | 489 | 84 | 0 | 0 | 489 | 786 | 24 | 4 | 0 | 0 | 24 | 17,663 |
| Bangladesh 2 | 27,270 | 1347 | 1139 | 0 | 0 | 1347 | 1246 | 112 | 103 | 2 | 5 | 114 | 27,055 |
| Bangladesh 3 | 19,435 | 201 | 199 | 38 | 0 | 236 | 918 | 14 | 14 | 2 | 0 | 17 | 20,100 |
| India 1 | 5,687 | 40 | 40 | 195 | 0 | 238 | 196 | 13 | 13 | 6 | 0 | 19 | 5,626 |
| India 2 | 44,405 | 13 | 1 | 13 | 0 | 26 | 579 | 0 | 0 | 0 | 0 | 0 | 44,958 |
| Nepal 1 | 22,869 | 237 | 12 | 158 | 0 | 238 | 708 | 9 | 0 | 6 | 0 | 9 | 23,330 |
| Nepal 2 | 29,002 | 65 | 0 | 6 | 38 | 71 | 957 | 1 | 0 | 0 | 1 | 1 | 29,887 |

BW: birthweight; GA: gestational age

**Annex S6: Covariate impacts on the recalibrated average birthweight by study**

|  | *Longitudinal training data from Nepal*  *Weight, grams*  *(standard error)* | *Bangladesh 1*  *Weight, grams*  *(standard error)* | *Bangladesh 2*  *Weight, grams*  *(standard error)* | *Bangladesh 3*  *Weight, grams*  *(standard error)* | *India 1*  *Weight, grams*  *(standard error)* |
| --- | --- | --- | --- | --- | --- |
| Maternal parity  (reference=previous birth) | -163.7 (49.6) | -165.1 (8.0) | -138.3 (5.5) | -156.9 (8.8) | -131.6 (9.0) |
| Newborn sex  (reference=female) | 50.6 (37.7) | 104.8 (5.4) | 93.1 (4.2) | 82.7 (6.7) | 97.1 (7.4) |
| Gestational age at delivery in weeks, centered at 40 weeks | 52.7 (7.9) | 43.7 (1.1) | 41.6(0.9) | 31.7 (1.3) | 31.7 (1.3) |
| Neonatal death  (reference = survives neonatal period or lost-to-follow-up) | N/A | -388.6 (12.9) | -366.0(11.0) | -450.9 (22.1) | -317.1 (30.3) |
| Maternal education in years | -0.5 (3.3) | 14.7 (0.7) | 14.8(0.6) | 14.8 (1.1) | 14.9 (0.9) |
| Maternal age in years | 10.7 (6.0) | 6.3 (0.7) | 5.4(0.5) | 4.1 (1.1) | 6.3 (1.1) |
| Multiple gestation (twins or triplets)  (reference = singleton) | N/A | -506.3 (24.3) | -556.5(18.6) | -654.9 (25.2) | -727.2 (42.3) |
| Intercept | 2745.1 (90.0) | 2393.8 (45.7) | 2466.0(39.6) | 2662.1 (22.1) | 2523.8 (29.2) |

|  | *Malawi*  *Weight, grams*  *(standard error)* | *Nepal 1*  *Weight, grams*  *(standard error)* | *Nepal 2*  *Weight, grams*  *(standard error)* | *Zambia*  *Weight, grams*  *(standard error)* |
| --- | --- | --- | --- | --- |
| Maternal parity  (reference=previous birth) | -146.5 (24.0) | -143.4 (6.9) | -106.2 (6.1) | -109.0 (7.6) |
| Newborn sex  (reference=female) | 80.1 (16.3) | 104.2 (5.3) | 111.1 (4.8) | 75.2 (5.5) |
| Gestational age at delivery in weeks, centered at 40 weeks | 107.3 (4.4) | 45.1 (1.1) | 32.5 (0.8) | 16.7 (0.8) |
| Neonatal death  (reference = survives neonatal period or lost-to-follow-up) | -290.3 (70.8) | -391.0 (20.9) | -341.8 (20.1) | -274.5 (34.3) |
| Maternal education in years | -3.3 (2.6) | 11.3 (0.6) | 12.6 (0.7) | N/A |
| Maternal age in years | 1.1 (1.6) | -1.0 (0.6) | 9.5 (0.6) | 3.8 (0.5) |
| Multiple gestation (twins or triplets)  (reference = singleton) | -607.2 (73.9) | -643.9 (22.3) | -650.5 (24.3) | -603.3 (19.3) |
| Intercept | 3062.2 (46.7) | 2735.8 (15.9) | 2569.1 (17.4) | 3031.4 (13.9) |

N/A: covariate was not collected or provided, recalibration was performed without the missing covariate.

**Table S7: Prevalence, neonatal mortality rate, and crude relative risk of neonatal mortality for six type categorization by study (relative risk reference: Term + Appropriate-for-gestational age)**

|  | **T+LGA** | | | **T+AGA** | | **T+SGA** | | |
| --- | --- | --- | --- | --- | --- | --- | --- | --- |
| **Study** | **Preval-ence** | **NN mortality rate** | **RR**  **(LL,UL)** | **Preval-ence** | **NN mortality rate** | **Preval-ence** | **NN mortality rate** | **RR**  **(LL, UL)** |
| Brazil | 12.0 | 0.0 | NA | 66.8 | 1.3 | 6.0 | 0 | NA |
| China | 5.6 | 5.3 | 0.7  (0.1,5.1) | 73.4 | 7.4 | 15.6 | 28.8 | 3.9  (2,7.3) |
| Bangladesh 1 | *Individual study estimates embargoed* | | | | | | | |
| Bangladesh 2 | *Individual study estimates embargoed* | | | | | | | |
| Bangladesh 3 | 1.5 | 7.2 | 0.5  (0.1,4.3) | 40.8 | 13.3 | 37.9 | 54.5 | 4.1  (3,5.5) |
| Burkina Faso | 1.0 | 0 | NA | 60.1 | 7.0 | 24.0 | 13.4 | 1.9  (0.7,5.1) |
| India 1 | 0.4 | 0 | NA | 36.4 | 14.7 | 49.5 | 29.8 | 2.0  (1.2,3.4) |
| India 2 | 0.8 | 5.5 | 0.9  (0.2,3.5) | 46.8 | 6.32 | 35.5 | 17.3 | 2.7  (2.2,3.4) |
| Malawi | 3.2 | 0 | NA | 66.2 | 10.6 | 18.8 | 27.0 | 2.5  (1.1,5.4) |
| Nepal 1 | 0.7 | 9.3 | 0.8  (0.1,4.8) | 39.3 | 11.6 | 41.5 | 29.0 | 2.5  (1.8,3.5) |
| Nepal 2 | 0.8 | 14.2 | 0.9  (0.2,3.6) | 42.0 | 14.6 | 41.6 | 29.6 | 2.0  (1.5,2.8) |
| Tanzania 1 | 5.5 | 17.4 | 1.2  (0.6,2.6) | 58.4 | 14.3 | 18.8 | 40.0 | 2.8  (2.0,4.0) |
| Tanzania 2 | 4.3 | 18.87 | 1.5 (0.2,11.2) | 69.9 | 12.85 | 20.5 | 35.86 | 2.8 (1.2,6.7) |
| Tanzania 3 | 4.1 | 5.9 | 0.9  (0.2,3.7) | 56.0 | 6.50 | 25.6 | 19.0 | 2.9  (1.8,4.7) |
| Zambia 1 | 5.9 | 7.5 | 0.9  (0.5,1.9) | 54.2 | 8.0 | 20.9 | 14.9 | 1.9  (1.2,2.8) |
| Zambia 2 | 4.2 | 9.4 | 1.3  (0.5,3.5) | 50.0 | 7.5 | 18.5 | 9.4 | 1.3  (0.7,2.2) |

NN: neonatal; RR: relative risk; LL: lower limit of the 95% confidence interval; UL: upper limit of the 95% confidence interval; T: term; PT: preterm; AGA: appropriate for gestational age; SGA: small for gestational age; LGA: large for gestational age; LBW: low birthweight; NA: no neonatal deaths; Excluded: lost-to-follow up during the NN period, improbable GA, improbable GA/BW combination (using imputed birthweight), missing baby gender, missing covariates to run BW imputation; LB: livebirths

### **Table S7 continued: Prevalence, neonatal mortality rate, and crude relative risk of neonatal mortality for six type categorization by study (relative risk reference: Term + Appropriate-for-gestational age)**

|  | **PT-LGA** | | | **PT-AGA** | | | **PT-SGA** | | |
| --- | --- | --- | --- | --- | --- | --- | --- | --- | --- |
| **Study** | **Preval-ence** | **NN mortality rate** | **RR**  **(LL,UL)** | **Preval-ence** | **NN mortality rate** | **RR**  **(LL,UL)** | **Preval-ence** | **NN mortality rate** | **RR**  **(LL,UL)** |
| Brazil | 2.9 | 58.3 | 54.6 (13.3,223.2) | 11.3 | 34.4 | 32.6 (9.5,112.6 | 1.3 | 163.7 | 155.2 (42.8,562.7) |
| China | 1.9 | 88.3 | 11.9 (5.2,27.1) | 3.3 | 84.4 | 11.4 (5.9,22.0) | 0.2 | <20 LBs | NA |
| Bangladesh 1 | *Individual study estimates embargoed* | | | | | | | | |
| Bangladesh 2 | *Individual study estimates embargoed* | | | | | | | | |
| Bangladesh 3 | 6.8 | 76.2 | 5.7  (4.1,8) | 11.0 | 88.4 | 6.7  (5,8.8) | 2.0 | 190.0 | 14.3 (9.9,20.6) |
| Burkina Faso | 4.2 | 13.1 | 1.7  (0.2,14.3) | 9.5 | 130.2 | 18.6 (8.9,39.1) | 1.3 | 41.0 | 5.9  (0.8,44.5) |
| India 1 | 3.0 | 108.3 | 7.3  (3.8,14.1) | 8.9 | 61.2 | 4.1  (2.3,7.5) | 1.8 | 147.6 | 10.0 (5.5,18.3) |
| India 2 | 1.7 | 14.36 | 2.3  (1.2,4.2) | 12.4 | 13.77 | 2.2  (1.6,2.9) | 2.7 | 65.3 | 10.3 (7.9,13.5) |
| Malawi | 1.6 | 261.5 | 24.1 (11.7,49.4) | 8.9 | 79.5 | 7.3  (3.6,15) | 1.3 | 43.5 | 3.6  (0.4,30.1) |
| Nepal 1 | 2.2 | 102.8 | 8.8  (6.2,12.6) | 13.3 | 51.4 | 4.4  (3.1,6.3) | 2.8 | 147.6 | 12.7 (9.0,17.8) |
| Nepal 2 | 3.9 | 114.5 | 7.9  (5.8,10.6) | 9.6 | 65.3 | 4.5  (3.6,5.6) | 2.2 | 121.0 | 8.3  (5.6,12.2) |
| Tanzania 1 | 8.1 | 27.8 | 1.9  (1.1,3.3) | 8.4 | 75.0 | 5.2  (3.6,7.6) | 0.8 | 210.3 | 14.7 (8.4,25.6) |
| Tanzania 2 | 0.1 | <20 LBs | NA | 3.8 | 282.61 | 22 (10.4,46.4) | 1.5 | <20 LBs | NA |
| Tanzania 3 | 7.8 | 10.9 | 1.7  (0.7,3.8) | 6.1 | 9.9 | 1.5  (0.6,3.9) | 0.3 | 40.0 | 5.3  (0.7,37.6) |
| Zambia 1 | 12.8 | 33.0 | 4.1  (3.2,5.3) | 5.9 | 35.6 | 4.4  (2.8,7) | 0.4 | 84.0 | 10.5  (5.2,21) |
| Zambia 2 | 17.8 | 16.4 | 2.2  (1.3,3.5) | 9.0 | 37.8 | 5.0  (3.2,8.0) | 0.6 | 70.2 | 9.3  (3.4,25.3) |

NN: neonatal; RR: relative risk; LL: lower limit of the 95% confidence interval; UL: upper limit of the 95% confidence interval; T: term; PT: preterm; AGA: appropriate for gestational age; SGA: small for gestational age; LGA: large for gestational age; LBW: low birthweight; NA: no neonatal deaths; Excluded: lost-to-follow up during the NN period, improbable GA, improbable GA/BW combination (using imputed birthweight), missing baby gender, missing covariates to run BW imputation; LB: livebirths

**Table S8: Prevalence, neonatal mortality rate, and crude relative risk of neonatal mortality for four type categorization by study (relative risk reference: Term + non-small for gestational age)**

|  | **T+nSGA** | | **T+SGA** | | | **PT + nSGA** | | | **PT + SGA** | | |
| --- | --- | --- | --- | --- | --- | --- | --- | --- | --- | --- | --- |
|  | **Preval-ence** | **NN mortality rate** | **Preval-ence** | **NN mortality rate** | **RR**  **(LL,UL)** | **Preval-ence** | **NN mortality rate** | **RR**  **(LL,UL)** | **Preval-ence** | **NN mortality rate** | **RR**  **(LL,UL)** |
| Brazil | 78.5 | 1.1 | 6.0 | 0 | NA | 14.2 | 39.2 | 43.8 (13.2,145.5) | 1.3 | 163.7 | 182.4 (50.3,661.5) |
| China | 79.0 | 7.3 | 15.6 | 28.8 | 3.9 (2.1,7.3) | 5.2 | 85.9 | 11.8 (6.7,20.7) | 0.2 | <20 LBs | NA |
| Bangladesh 1 | *Individual study estimates embargoed* | | | | | | | | | | |
| Bangladesh 2 | *Individual study estimates embargoed* | | | | | | | | | | |
| Bangladesh 3 | 42.4 | 13.1 | 37.9 | 54.5 | 4.2 (3.1,5.6) | 17.8 | 83.7 | 6.4  (5,8.3) | 2.0 | 190.0 | 14.5 (10.2,20.7) |
| Burkina Faso | 61.0 | 6.8 | 24.1 | 13.4 | 2.0 (0.7,5.2) | 13.8 | 94.1 | 13.7 (6.6,28.8) | 1.1 | 41.0 | 6.0 (0.8,45.5) |
| India 1 | 36.8 | 14.5 | 49.5 | 29.8 | 2.1 (1.2,3.5) | 11.9 | 73.1 | 5.1  (3,8.5) | 1.8 | 147.6 | 10.2 (5.6,18.6) |
| India 2 | 47.6 | 6.3 | 35.5 | 17.3 | 2.7 (2.2,3.4) | 14.1 | 13.8 | 2.2  (1.7,2.9) | 2.7 | 65.3 | 10.4 (7.9,13.6) |
| Malawi | 69.5 | 10.1 | 18.8 | 27.0 | 2.7 (1.2,5.8) | 10.4 | 107.1 | 10.6 (5.8,19.5) | 1.3 | 43.5 | 3.8 (0.5,32.4) |
| Nepal 1 | 40.1 | 11.6 | 41.6 | 29.0 | 2.5 (1.8,3.5) | 15.5 | 58.7 | 5.1  (3.7,6.9) | 2.8 | 147.6 | 12.7 (9.1,17.8) |
| Nepal 2 | 42.7 | 14.6 | 41.5 | 29.6 | 2.0 (1.5,2.8) | 13.6 | 79.6 | 5.5  (4.3,6.9) | 2.2 | 121.0 | 8.3 (5.6,12.3) |
| Tanzania 1 | 63.9 | 14.7 | 18.8 | 39.8 | 2.7 (1.9,3.9) | 16.5 | 51.9 | 3.6  (2.5,5.0) | 0.8 | 206.9 | 14.4 (8.3,25.0) |
| Tanzania 2 | 74.2 | 13.2 | 20.5 | 35.9 | 2.7 (1.2,6.4) | 3.8 | 276.6 | 20.9 (10.1,43.3) | 1.5 | <20 LBs | NA |
| Tanzania 3 | 60.3 | 6.4 | 25.4 | 19.0 | 3.0  (1.9,4.7) | 14.0 | 10.3 | 1.6 (0.8,3.1) | 0.3 | 34.5 | 5.3 (0.7,39.1) |
| Zambia 1 | 60.1 | 8.0 | 20.8 | 14.9 | 1.9 (1.2,2.9) | 18.7 | 33.8 | 4.3 (3.3,5.4) | 0.4 | 84.0 | 10.5 (5.3,21) |
| Zambia 2 | 54.2 | 7.7 | 18.5 | 9.4 | 1.2 (0.7,2.2) | 26.7 | 23.6 | 3.1 (2.1,4.5) | 0.6 | 70.2 | 9.1 (3.4,24.7) |

NN: neonatal; RR: relative risk; LL: lower limit of the 95% confidence interval; UL: upper limit of the 95% confidence interval; T: term; PT: preterm; AGA: appropriate for gestational age; SGA: small for gestational age; LGA: large for gestational age; LB: livebirths; nSGA: non-SGA (AGA and LGA combined)

**Table S9: Prevalence, neonatal mortality rate, and crude relative risk of neonatal mortality for nine type categorization by study (relative risk reference: Term + Appropriate-for-gestational age + Non-low birthweight)**

|  | **T + LGA + non-LBW** | | | **T + AGA + non-LBW** | | **T + AGA + LBW** | | | **T + SGA + non-LBW** | | |
| --- | --- | --- | --- | --- | --- | --- | --- | --- | --- | --- | --- |
| **Study** | **Preval-ence** | **NN mortality rate** | **RR**  **(LL,UL)** | **Preval-ence** | **NN mortality rate** | **Preval-ence** | **NN mortality rate** | **RR**  **(LL,UL)** | **Preval-ence** | **NN mortality rate** | **RR**  **(LL,UL)** |
| Brazil | 11.7 | 0.0 | NA | 66.4 | 1.3 | 0.3 | 0.0 | NA | 3.7 | 0.0 | NA |
| China | 5.6 | 5.3 | 0.7 (0.1,5.1) | 73.3 | 7.5 | 0.1 | <20 LB | NA | 12.6 | 21.2 | 2.8 (1.4,5.9) |
| Bangladesh 1 | *Individual study estimates embargoed* | | | | | | | | | | |
| Bangladesh 2 | *Individual study estimates embargoed* | | | | | | | | | | |
| Bangladesh 3 | 1.5 | 7.2 | 0.5 (0.1,4.3) | 40.3 | 13.0 | 0.5 | 42.3 | 3.1 (0.8,12.1) | 19.5 | 30.9 | 2.4 (1.6,3.5) |
| Burkina Faso | 1.0 | 0 | NA | 59.6 | 7.0 | 0.4 | <20 LB | NA | 15.6 | 11.7 | 1.7 (0.5,5.3) |
| India 1 | 0.4 | 0 | NA | 36 | 14.5 | 0.4 | 0 | NA | 27.9 | 20.3 | 1.4 (0.8,2.6) |
| India 2 | 0.8 | 5.5 | 0.9 (0.2,3.6) | 46.3 | 6.19 | 0.5 | 19.2 | 3.1 (1.2,8.3) | 19.8 | 8.9 | 1.4 (1.1,1.9) |
| Malawi | 3.2 | 0 | NA | 65.7 | 10.7 | 0.6 | <20 LB | NA | 11.5 | 27.2 | 2.4 (1,5.9) |
| Nepal 1 | 0.7 | 9.4 | 0.8 (0.1,5.1) | 38.4 | 11.1 | 0.9 | 35.5 | 3.2 (1.1,8.8) | 23 | 15.8 | 1.4 (0.9,2.3) |
| Nepal 2 | 0.8 | 14.2 | 1.0 (0.2,3.7) | 41.3 | 14.1 | 0.7 | 43.2 | 2.9 (0.9,9.8) | 23.4 | 23.6 | 1.7 (1.2,2.3) |
| Tanzania 1 | 5.5 | 17.4 | 1.2 (0.6,2.6) | 58.3 | 14.4 | 0.1 | NA | NA | 14.6 | 29.8 | 2.1 (1.4,3.2) |
| Tanzania 2 | 4.3 | 18.9 | 1.5 (0.2,11.1) | 69.6 | 12.9 | 0.2 | <20 LB | NA | 14.6 | 22.3 | 1.7 (0.6,5.4) |
| Tanzania 3 | 4.2 | 5.8 | 0.9 (0.2,3.7) | 56.0 | 6.5 | 0.0 | NA | NA | 18.9 | 13.5 | 2.1 (1.2,3.6) |
| Zambia 1 | 5.9 | 7.5 | 0.9 (0.5,1.9) | 53.9 | 7.9 | 0.3 | 21.2 | 2.4 (0.4,16.4) | 15.4 | 11.3 | 1.4 (0.9,2.2) |
| Zambia 2 | 4.2 | 9.4 | 1.3 (0.5,3.6) | 49.8 | 7.5 | 0.2 | 0 | NA | 14.2 | 4.9 | 0.7 (0.3,1.5) |

NN: neonatal; RR: relative risk; LL: lower limit of the 95% confidence interval; UL: upper limit of the 95% confidence interval; T: term; PT: preterm; AGA: appropriate for gestational age; SGA: small for gestational age; LGA: large for gestational age; LBW: low birthweight; NA: no neonatal deaths; Excluded: lost-to-follow up during the NN period, improbable GA, improbable GA/BW combination (using imputed birthweight), missing baby gender, missing covariates to run BW imputation; LB: livebirths

### **Table S9 continued: Prevalence, neonatal mortality rate, and crude relative risk of neonatal mortality for nine type categorization by study (relative risk reference: Term + Appropriate-for-gestational age + Non-low birthweight)**

|  | **T + SGA + LBW** | | | **PT + LGA + non-LBW** | | | **PT + LGA + LBW** | | |
| --- | --- | --- | --- | --- | --- | --- | --- | --- | --- |
|  | **Preval-ence** | **NN mortality rate** | **RR**  **(LL,UL)** | **Preval-ence** | **NN mortality rate** | **RR**  **(LL,UL)** | **Preval-ence** | **NN mortality rate** | **RR**  **(LL,UL)** |
| Brazil | 2.3 | 0 | NA | 2.2 | 0.0 | NA | 0.7 | 229.2 | 207 (53.1,809.7) |
| China | 2.9 | 61.4 | 8.1 (3.1,21.5) | 1.8 | 41.0 | 5.5 (1.6,18.4) | 0.2 | <20 LB | NA |
| Bangladesh 1 | *Individual study estimates embargoed* | | | | | | | | |
| Bangladesh 2 | *Individual study estimates embargoed* | | | | | | | | |
| Bangladesh 3 | 18.3 | 79.5 | 6.2  (4.6,8.3) | 5.4 | 22.0 | 1.7 (0.9,3.1) | 1.4 | 289.5 | 22.3 (15.5,32.1) |
| Burkina Faso | 8.3 | 16.7 | 2.4  (0.6,8.7) | 3.2 | 0 | NA | 1.0 | 45.9 | 14.0  (2.4,81) |
| India 1 | 21.6 | 42.0 | 2.9  (1.7,4.9) | 2.2 | 18.2 | 1.1 (0.1,9.7) | 0.8 | 341.8 | 23.9 (13.8,41.4) |
| India 2 | 15.7 | 27.9 | 4.5  (3.6,5.6) | 1.6 | 8.5 | 1.4 (0.6,3.1) | 0.1 | 82.0 | 13.2 (5.6,31.2) |
| Malawi | 7.3 | 26.6 | 2.4 (0.5,10.9) | 1.1 | 0 | NA | 0.5 | <20 LB | NA |
| Nepal 1 | 18.5 | 45.5 | 4.1  (2.9,5.9) | 1.8 | 13.5 | 1.1 (0.3,4.2) | 0.4 | 496.8 | 45.0  (32.6,62.1) |
| Nepal 2 | 18.2 | 37.3 | 2.6  (1.9,3.7) | 2.9 | 55.8 | 3.9 (2.9,5.4) | 1.0 | 291.5 | 20.6  (14,30.3) |
| Tanzania 1 | 4.2 | 75.5 | 5.3  (3.3,8.3) | 7.5 | 13.2 | 0.9 (0.4,2) | 0.6 | 212.7 | 14.8 (7.9,27.6) |
| Tanzania 2 | 5.9 | 69.4 | 5.4 (1.9,15.1) | 0.0 | <20 LB | NA | 0.1 | <20 LB | NA |
| Tanzania 3 | 6.5 | 35.5 | 5.5  (3.1,9.6) | 7.5 | 4.8 | 0.7 (0.2,2.4) | 0.3 | 166.7 | 25.6 (9.8,67.2) |
| Zambia 1 | 5.5 | 25.2 | 3.1  (1.7,5.7) | 12.0 | 23.7 | 3.0 (2.1,4.2) | 0.8 | 173.6 | 21.7  (13,36.3) |
| Zambia 2 | 4.3 | 24.0 | 3.1  (1.6,6.2) | 16.2 | 9.4 | 1.3 (0.7,2.3) | 1.5 | 92.1 | 12.4 (6.8,22.7) |

NN: neonatal; RR: relative risk; LL: lower limit of the 95% confidence interval; UL: upper limit of the 95% confidence interval; T: term; PT: preterm; AGA: appropriate for gestational age; SGA: small for gestational age; LGA: large for gestational age; LBW: low birthweight; NA: no neonatal deaths; Excluded: lost-to-follow up during the NN period, improbable GA, improbable GA/BW combination (using imputed birthweight), missing baby gender, missing covariates to run BW imputation; LB: livebirths

**Table S9 continued: Prevalence, neonatal mortality rate, and crude relative risk of neonatal mortality for nine type categorization by study (relative risk reference: Term + Appropriate-for-gestational age + Non-low birthweight)**

|  | **PT + AGA + non-LBW** | | | **PT + AGA + LBW** | | | **PT + SGA + LBW** | | |
| --- | --- | --- | --- | --- | --- | --- | --- | --- | --- |
|  | **Preval-ence** | **NN mortality rate** | **RR**  **(LL,UL)** | **Preval-ence** | **NN mortality rate** | **RR**  **(LL,UL)** | **Preval-ence** | **NN mortality rate** | **RR**  **(LL,UL)** |
| Brazil | 5.5 | 0.0 | **NA** | 5.8 | 66.6 | 61.0 (17.8,209.1) | 1.3 | 163.7 | 137.3 (37.0,509.4) |
| China | 2.0 | 20.9 | 2.7 (0.5,15.1) | 1.2 | 189.0 | 25.4  (13,49.7) | 0.2 | <20 LB | **NA** |
| Bangladesh 1 | *Individual study estimates embargoed* | | | | | | | | |
| Bangladesh 2 | *Individual study estimates embargoed* | | | | | | | | |
| Bangladesh 3 | 6.0 | 22.5 | 1.7  (1,2.9) | 5.0 | 166.5 | 12.9 (9.5,17.5) | 2.0 | 190.1 | 14.7 (10.3,21) |
| Burkina Faso | 4.2 | 0 | NA | 5.4 | 227.1 | 32.7 (15.6,68.3) | 1.3 | 40.98 | 5.9 (0.8,44.6) |
| India 1 | 4.4 | 24.2 | 1.6 (0.5,5.1) | 4.5 | 97.5 | 6.8  (3.8,12.2) | 1.8 | 147.57 | 10.3 (5.6,18.7) |
| India 2 | 7.8 | 8.0 | 1.3 (0.9,1.9) | 4.7 | 23.4 | 3.8  (2.7,5.2) | 2.7 | 65.3 | 10.5  (8,13.8) |
| Malawi | 4.5 | 19.7 | 1.7 (0.3,10.9) | 4.4 | 141.8 | 12.9 (6.5,25.6) | 1.3 | 43.5 | 3.5 (0.4,29.4) |
| Nepal 1 | 8.0 | 14.8 | 1.3 (0.8,2.3) | 5.3 | 106.7 | 9.7  (6.6,14.2) | 2.8 | 147.6 | 13.4 (9.2,19.4) |
| Nepal 2 | 4.9 | 19.6 | 1.4 (0.9,2.2) | 4.7 | 113.1 | 8  (6.4,10.1) | 2.2 | 121.0 | 8.6 (5.8,12.6) |
| Tanzania 1 | 5.8 | 22.7 | 1.6 (0.8,3.1) | 2.6 | 190.8 | 13.3  (9,19.5) | 0.8 | 210.3 | 14.6 (8.4,25.6) |
| Tanzania 2 | 1.2 | <20 LB | NA | 2.5 | 419.4 | 32.5 (15.9,66.7) | 1.5 | <20 LB | NA |
| Tanzania 3 | 4.8 | 10.1 | 1.6 (0.6,4.4) | 1.3 | 9.01 | 1.4  (0.2,10.1) | 0.4 | 34.5 | 5.3 (0.7,37.6) |
| Zambia 1 | 4.1 | 19.6 | 2.5 (1.4,4.4) | 1.7 | 30.5 | 9.2  (5.6,15.2) | 0.4 | 84.0 | 10.6 (5.3,21.2) |
| Zambia 2 | 6.6 | 15.9 | 2.1  (1.1,4.3) | 2.4 | 97.0 | 13.0 (7.9,21.6) | 0.6 | 70.2 | 9.5 (3.5,25.6) |

NN: neonatal; RR: relative risk; LL: lower limit of the 95% confidence interval; UL: upper limit of the 95% confidence interval; T: term; PT: preterm; AGA: appropriate for gestational age; SGA: small for gestational age; LGA: large for gestational age; LBW: low birthweight; NA: no neonatal deaths; Excluded: lost-to-follow up during the NN period, improbable GA, improbable GA/BW combination (using imputed birthweight), missing baby gender, missing covariates to run BW imputation; LB: livebirths

Table S10: Comparison of median birthweight (1) measured within 72 hours, versus (2) using recalibration and/or imputation method by type of imputation method and median time of birthweight measured.

| **Study** | **Imputation method** | **Median time of birthweight measurement*** | **Median birthweight measured <72 hours** | **Median birthweight using recalibration/ imputation** | **∆ grams** |
| --- | --- | --- | --- | --- | --- |
| China | Imputation | 97% measured <24h | 3200 | 3200 | 0 |
| Brazil | Imputation | 100% measured <24h | 3203 | 3200 | 3 |
| Burkina Faso | Imputation | 100% measured <24h | 2920 | 2900 | 20 |
| Zambia 2 | Imputation | 95% measured <24h | 3000 | 3000 | 0 |
| Bangladesh 3 | Imputation/ recalibration | 7.6 hours | 2720 | 2770 | -50 |
| Malawi | Imputation/ recalibration | 19.3 hours | 3000 | 2999 | 1 |
| India 1 | Imputation/ recalibration | 23.4 hours | 2665 | 2705 | -40 |
| Nepal 1 | Imputation/ recalibration | 24 hours | 2700 | 2740 | -40 |
| Nepal 2 | Imputation/ recalibration | 12.8 hours | 2740 | 2761 | -21 |
| Zambia 1 | Imputation/ recalibration | 24 hours | 3000 | 3078 | -78 |

***Among those with non-missing BW**

Table S11: Comparison of neonatal mortality rates and relative risk for four-type categorization using birthweights (1) measured within 72 hours, versus (2) using recalibration and/or imputation method.


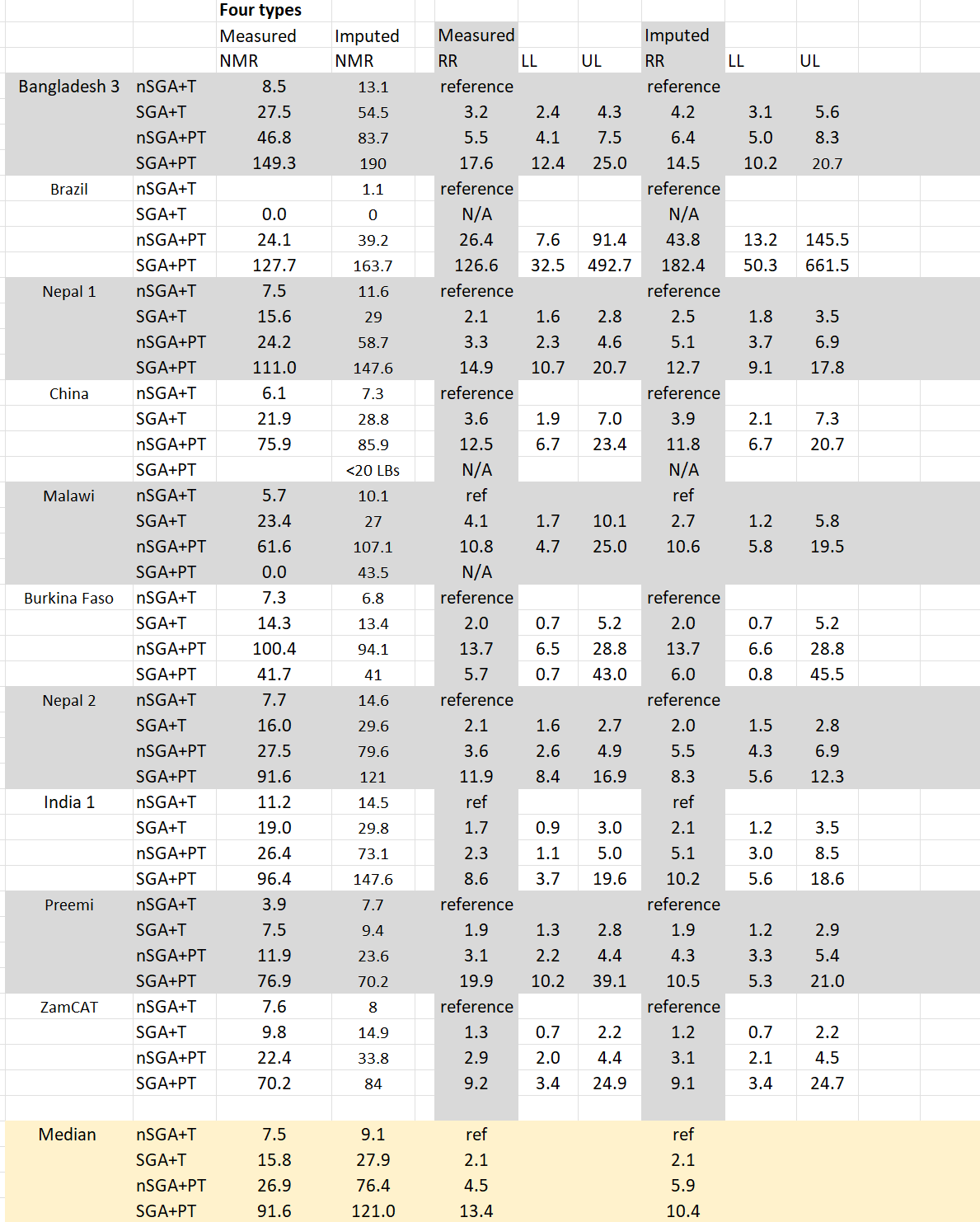


NMR: neonatal mortality rate, RR: relative risk, LL: 95% confidence interval - lower limit, UL: 95% confidence interval - upper limit, T: Term, PT: Preterm, nSGA: non-small for gestational age (AGA and LGA combined), SGA: small for gestational age


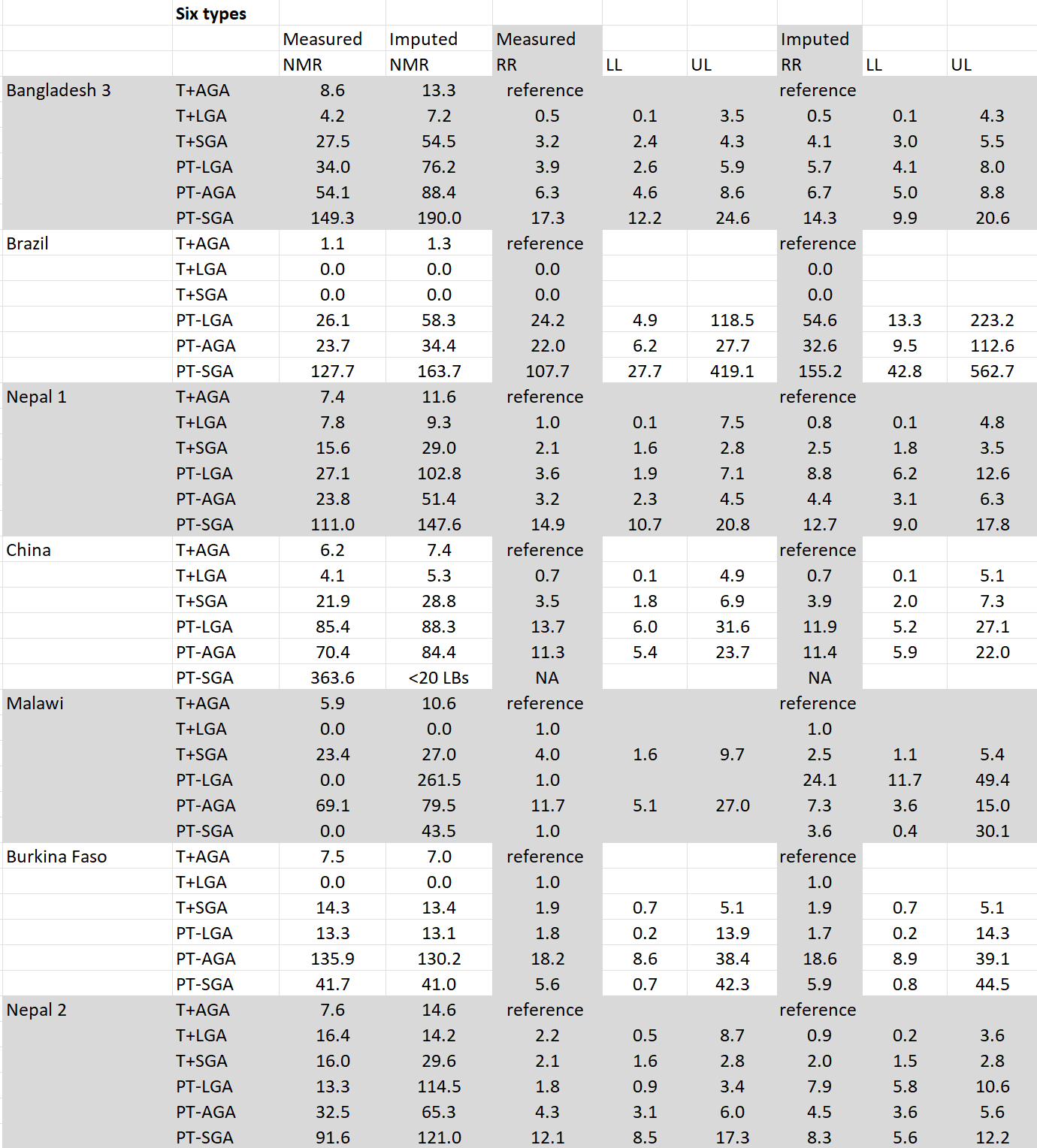


Table S12: Comparison of neonatal mortality rates and relative risk for six-type categorization using birthweights (1) measured within 72 hours, versus (2) using recalibration and/or imputation method.


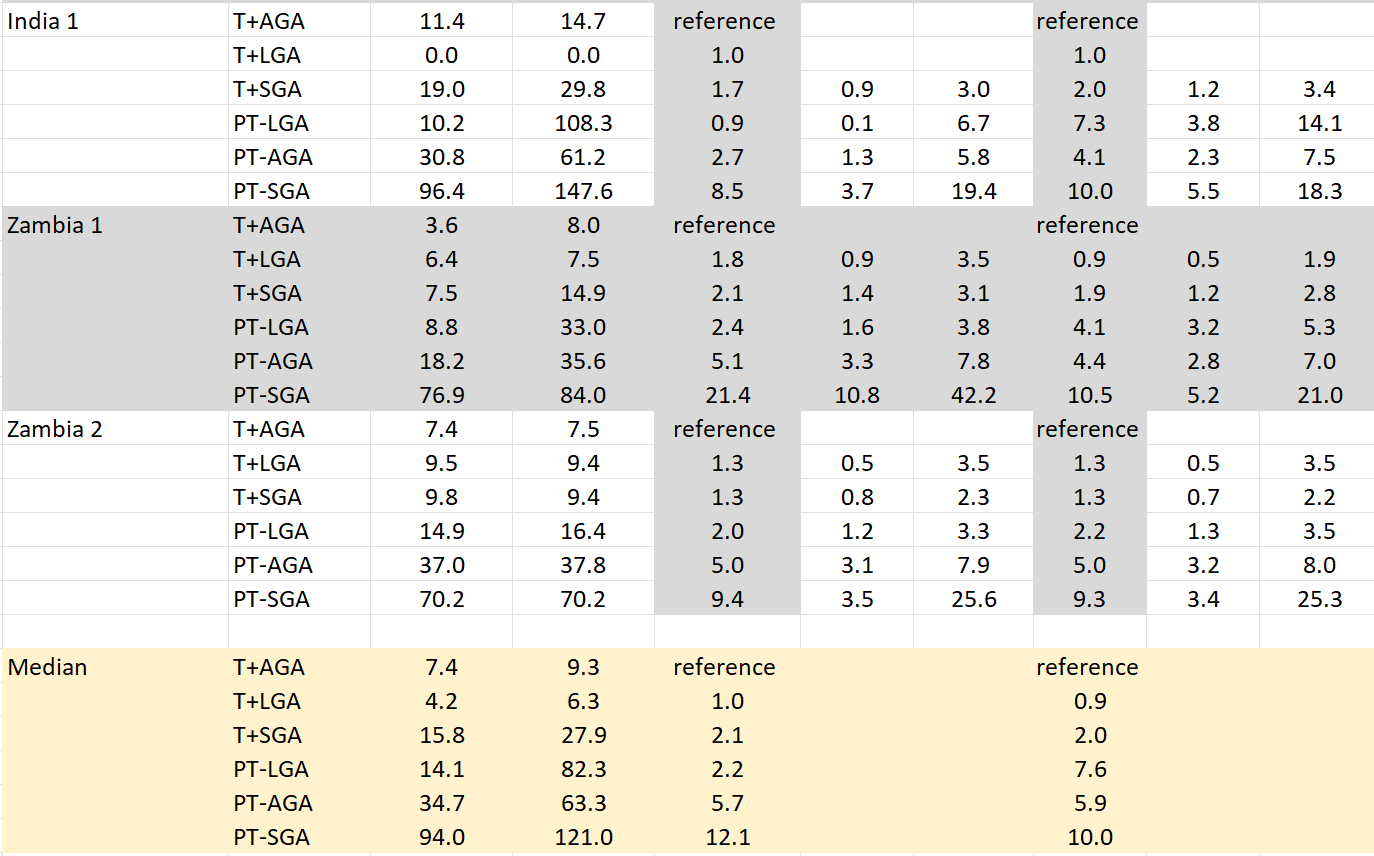
NMR: neonatal mortality rate, RR: relative risk, LL: 95% confidence interval - lower limit, UL: 95% confidence interval - upper limit; T: Term, PT: Preterm, AGA: appropriate for gestational age, SGA: small for gestational age, LGA: large for gestational age

**Supplementary references:**

1. Elm E von, Altman DG, Egger M, Pocock SJ, Gøtzsche PC, Vandenbroucke JP. The Strengthening the Reporting of Observational Studies in Epidemiology (STROBE) statement: guidelines for reporting observational studies. The Lancet. 2007 Oct 20;370(9596):1453–7.

2. Zeng L, Dibley MJ, Cheng Y, Dang S, Chang S, Kong L, et al. Impact of micronutrient supplementation during pregnancy on birth weight, duration of gestation, and perinatal mortality in rural western China: double blind cluster randomised controlled trial. BMJ. 2008 Nov 7;337:a2001.

3. Hallal PC, Bertoldi AD, Domingues MR, da Silveira MF, Demarco FF, da Silva ICM, et al. Cohort Profile: The 2015 Pelotas (Brazil) Birth Cohort Study. Int J Epidemiol. 2018 Aug 1;47(4):1048–1048h.

4. Roberfroid D, Huybregts L, Lanou H, Henry MC, Meda N, Menten J, et al. Effects of maternal multiple micronutrient supplementation on fetal growth: a double-blind randomized controlled trial in rural Burkina Faso. Am J Clin Nutr. 2008 Nov;88(5):1330–40.

5. MISAME Study Group. Prenatal food supplementation fortified with multiple micronutrients increases birth length: a randomized controlled trial in rural Burkina Faso. The American Journal of Clinical Nutrition. 2009 Dec 1;90(6):1593–600.

6. Ashorn P, Alho L, Ashorn U, Cheung YB, Dewey KG, Harjunmaa U, et al. The impact of lipid-based nutrient supplement provision to pregnant women on newborn size in rural Malawi: a randomized controlled trial. The American Journal of Clinical Nutrition. 2015 Feb 1;101(2):387–97.

7. Luntamo M, Kulmala T, Mbewe B, Cheung YB, Maleta K, Ashorn P. Effect of Repeated Treatment of Pregnant Women with Sulfadoxine-Pyrimethamine and Azithromycin on Preterm Delivery in Malawi: A Randomized Controlled Trial. Am J Trop Med Hyg. 2010 Dec 6;83(6):1212–20.

8. Fawzi WW, Msamanga GI, Urassa W, Hertzmark E, Petraro P, Willett WC, et al. Vitamins and Perinatal Outcomes among HIV-Negative Women in Tanzania. N Engl J Med. 2007 Apr 5;356(14):1423–31.

9. Hjort L, Møller SL, Minja D, Msemo O, Nielsen BB, Christensen DL, et al. FOETAL for NCD—FOetal Exposure and Epidemiological Transitions: the role of Anaemia in early Life for Non-Communicable Diseases in later life: a prospective preconception study in rural Tanzania. BMJ Open. 2019 May 1;9(5):e024861.

10. Schmiegelow C, Scheike T, Oesterholt M, Minja D, Pehrson C, Magistrado P, et al. Development of a Fetal Weight Chart Using Serial Trans-Abdominal Ultrasound in an East African Population: A Longitudinal Observational Study. PLOS ONE. 2012 Sep 21;7(9):e44773.

11. Masanja H, Smith ER, Muhihi A, Briegleb C, Mshamu S, Ruben J, et al. Effect of neonatal vitamin A supplementation on mortality in infants in Tanzania (Neovita): a randomised, double-blind, placebo-controlled trial. Lancet. 2015 Apr 4;385(9975):1324–32.

12. Effectiveness of 4% chlorhexidine umbilical cord care on neonatal mortality in Southern Province, Zambia (ZamCAT): a cluster-randomised controlled trial - ClinicalKey [Internet]. [cited 2022 Jun 13]. Available from: https://www-clinicalkey-com.proxy1.library.jhu.edu/#!/content/playContent/1-s2.0-S2214109X16302157?returnurl=https:%2F%2Flinkinghub.elsevier.com%2Fretrieve%2Fpii%2FS2214109X16302157%3Fshowall%3Dtrue&referrer=https:%2F%2Fpubmed.ncbi.nlm.nih.gov%2F

13. Tembo T, Koyuncu A, Zhuo H, Mwendafilumba M, Manasyan A. The association of maternal age with adverse neonatal outcomes in Lusaka, Zambia: a prospective cohort study. BMC Pregnancy and Childbirth. 2020 Nov 11;20(1):684.

14. Klemm RDW, Merrill RD, Wu L, Shamim AA, Ali H, Labrique A, et al. Low-birthweight rates higher among Bangladeshi neonates measured during active birth surveillance compared to national survey data. Matern Child Nutr. 2015 Oct;11(4):583–94.

15. West KP, Shamim AA, Mehra S, Labrique AB, Ali H, Shaikh S, et al. Effect of maternal multiple micronutrient vs iron-folic acid supplementation on infant mortality and adverse birth outcomes in rural Bangladesh: the JiVitA-3 randomized trial. JAMA. 2014 Dec 24;312(24):2649–58.

16. Saha SK, Schrag SJ, El Arifeen S, Mullany LC, Shahidul Islam M, Shang N, et al. Causes and incidence of community-acquired serious infections among young children in south Asia (ANISA): an observational cohort study. The Lancet. 2018 Jul 14;392(10142):145–59.

17. Mitra DK, Mahmud A, Begum N, Rafiqullah I, Roy A, Moin SMI, et al. Implementation of the ANISA Protocol in Sylhet, Bangladesh: Challenges and Solutions. Pediatr Infect Dis J. 2016 May;35(5 Suppl 1):S55-59.

18. Rahmathullah L, Tielsch JM, Thulasiraj RD, Katz J, Coles C, Devi S, et al. Impact of supplementing newborn infants with vitamin A on early infant mortality: community based randomised trial in southern India. BMJ. 2003 Aug 2;327(7409):254.

19. Mazumder S, Taneja S, Bhatia K, Yoshida S, Kaur J, Dube B, et al. Efficacy of early neonatal supplementation with vitamin A to reduce mortality in infancy in Haryana, India (Neovita): a randomised, double-blind, placebo-controlled trial. The Lancet. 2015 Apr 4;385(9975):1333–42.

20. Tielsch JM, Darmstadt GL, Mullany LC, Khatry SK, Katz J, LeClerq SC, et al. Impact of Newborn Skin-Cleansing With Chlorhexidine on Neonatal Mortality in Southern Nepal: A Community-Based, Cluster-Randomized Trial. Pediatrics. 2007 Feb;119(2):e330–40.

21. Mullany LC, Darmstadt GL, Khatry SK, Katz J, LeClerq SC, Shrestha S, et al. Topical applications of chlorhexidine to the umbilical cord for prevention of omphalitis and neonatal mortality in southern Nepal: a community-based, cluster-randomised trial. The Lancet. 2006 Mar 18;367(9514):910–8.

22. Impact of Sunflower Seed Oil Massage on Neonatal Mortality and Morbidity in Nepal (NOMS) [Internet]. Available from: https://clinicaltrials.gov/ct2/show/NCT01177111

1. Excludes lost-to-follow-up [↑](#footnote-ref-1)
2. Among those with measured birthweight [↑](#footnote-ref-2)
3. Prior to birthweight imputation, excludes LTFU [↑](#footnote-ref-3)
4. Sub-cohort with more complete GA [↑](#footnote-ref-4)
5. No missing birthweight in this study, missing due to GA [↑](#footnote-ref-5)
6. Livebirths without complete covariates to conduct imputation were excluded [↑](#footnote-ref-6)
7. Includes birthweights measured >72 hours for studies with no imputation [↑](#footnote-ref-7)
8. The total exclusion number is not additive of the reasons for exclusion since they are not mutually exclusive. [↑](#footnote-ref-8)
